# Gliding Associated Proteins Play Essential Roles during the Formation of the Inner Membrane Complex of *Toxoplasma gondii*

**DOI:** 10.1371/journal.ppat.1005403

**Published:** 2016-02-04

**Authors:** Clare R. Harding, Saskia Egarter, Matthew Gow, Elena Jiménez-Ruiz, David J. P. Ferguson, Markus Meissner

**Affiliations:** 1 Wellcome Trust Centre for Molecular Parasitology, Institute of Infection, Immunity & Inflammation, College of Medical, Veterinary and Life Sciences, University of Glasgow, Glasgow, United Kingdom; 2 Nuffield Department of Clinical Laboratory Science, Oxford University, Oxford, United Kingdom; Boston College, UNITED STATES

## Abstract

The inner membrane complex (IMC) of apicomplexan parasites is a specialised structure localised beneath the parasite’s plasma membrane, and is important for parasite stability and intracellular replication. Furthermore, it serves as an anchor for the myosin A motor complex, termed the glideosome. While the role of this protein complex in parasite motility and host cell invasion has been well described, additional roles during the asexual life cycle are unknown. Here, we demonstrate that core elements of the glideosome, the gliding associated proteins GAP40 and GAP50 as well as members of the GAPM family, have critical roles in the biogenesis of the IMC during intracellular replication. Deletion or disruption of these genes resulted in the rapid collapse of developing parasites after initiation of the cell cycle and led to redistribution of other glideosome components.

## Author Summary

*Toxoplasma gondii* is an important parasite of humans and animals that must actively invade host cells in order to replicate. Beneath the surface of the parasite lies the inner membrane complex (IMC) which is important in maintaining the stability of the parasite, as well as acting as a base for a protein complex known as the glideosome. This assembly of proteins has an important role in allowing the parasite to invade host cells. Here, we examined the function of proteins known to be part of the glideosome, GAP40, GAP50 and five proteins of the GAPM family. We found that in the absence of GAP40 or GAP50, the parasite is able to start replication but is unable to complete it, suggesting that these proteins have a structural role in maintaining the stability of the developing IMC during replication. We also saw that disruption of some members of the GAPM protein family led to a loss of parasite structure. Our study demonstrates that some components of the glideosome have multiple roles in *T*. *gondii* biology and gives us new insights into how cells are constructed during parasite replication.

## Introduction

*Toxoplasma gondii* is a promiscuous parasite able to invade and replicate within any nucleated cell of warm-blooded animals. Although usually asymptomatic, infection can lead to serious complications in the immunocompromised and pregnant women and is a common cause of eye disease in South America. *T*. *gondii*, like other apicomplexa species, contains a unique structure known as the inner membrane complex (IMC). This lies beneath the plasma membrane to maintain the structure of the parasite and acts as a scaffold during cell division [1–3]. The IMC consists of alveoli supported on the cytoplasmic face by a highly organised network of intermediate filament-like proteins (also known as ‘alveolins’) termed the subpellicular network (SPN) [2,4,5] and by interactions with the subpellicular microtubule cytoskeleton [6,7]. These interactions are thought to be mediated by 9 nm intramembranous particles (IMPs) found with distinct periodicity on all four faces of the alveolar membranes [7,8].

In addition, the IMC is critical for the anchorage and stabilisation of the glideosome [9], which fulfils an important role during the invasion of the host cell [10–12]. Assembly of this multi-subunit complex occurs in two steps [13]. The gliding associated proteins GAP40 and 50 are transported to the IMC via the secretory pathway [14,15] and are present in the IMC of daughter cells early during endodyogeny [15,16]. In contrast, the core motor complex consisting of MyoA, MLC1 and GAP45 are initially assembled as a soluble complex in the cytoplasm [13] and N-terminal acylation of GAP45 anchors the pre-complex to the parasite plasma membrane [17]. This pre-complex subsequently associates with GAP50 and GAP40 in mature parasites, forming a stable, immobilised complex anchored between the plasma membrane and the IMC [13,16].

While the glideosome is known to be important for motility, invasion and egress, there is currently little evidence for a role of its components in replication [16,18]. While it has been hypothesised that MyoA is involved in Rab11A and Rab11B-dependent vesicular transport [19,20], recent studies using a conditional knockout system have demonstrated that neither MyoA, MLC1, GAP45 nor actin are directly involved in IMC biogenesis [11,21].

Here, we re-addressed the role of the individual components of the glideosome during intracellular development of the parasite and identified GAP40, GAP50 and the GAPM proteins as critical factors for the biogenesis of nascent IMC, presumably acting as structural proteins in concert with GAP45. Together, our results expand the functions assigned to the glideosome establishing it as a target to interfere with all steps during the asexual life cycle (gliding motility, host cell invasion, egress and intracellular replication).

## Results

### Conditional deletion of *gap40* and *gap50*

**Fig 1:**
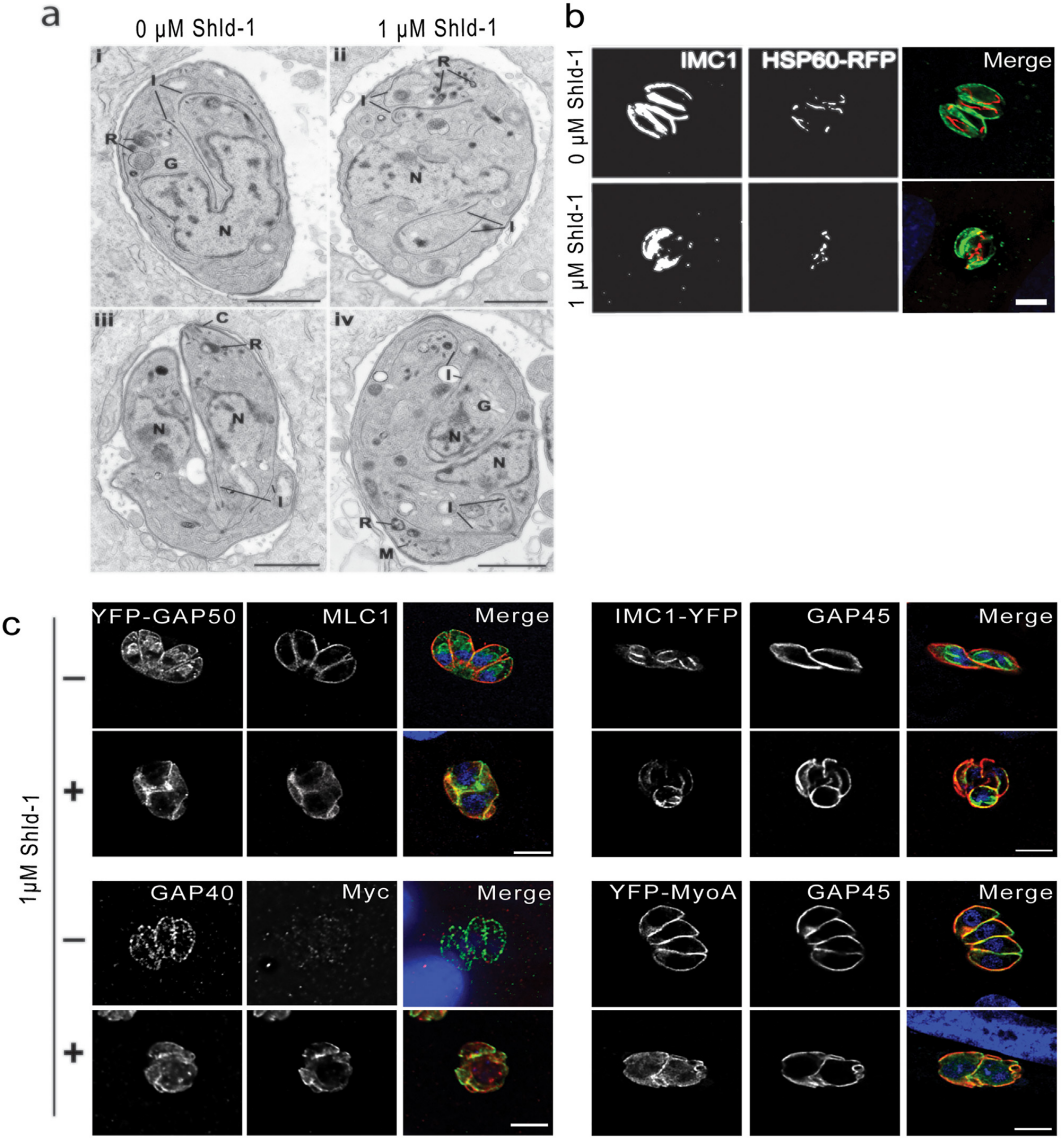
Overexpression of MyoA_tail_ leads to morphological disruption of the parasite’s structure. MyoA_tail_-expressing parasites were treated with 1 μM Shld-1 and fixed after 24 h. **(a)** Electron microscopy of ddMyc-MyoA_tail_ untreated (i, iii) or treated with 1 μM Shld-1 (ii, iii) for 24 h. (**i**) Longitudinal section of a cell undergoing endodyogeny showing the conical shaped IMC (I) of the two daughters enclosing the dividing nucleus (N). (**ii**) Section showing an enlarged nucleus (N) with the cytoplasm containing partially disorganised plates of IMC (I). (**iii**) Late stage in endodyogeny showing the folding of the plasmalemma to form the pellicle of the daughters. (**iv**) Section showing a parasite with multiple nuclei (N) and the cytoplasm contain a number of partially formed daughters. C—conoid; G—Golgi body; I—IMC; R—rhoptries; M—Microneme. Scale bar 1 μm. (**b**) MyoA_tail_-expressing parasites co-expressing mitochondrial marker *hsp60-rfp* were stained using anti-IMC1. Overexpression of MyoA_tail_ resulted in an alteration of mitochondrial morphology. (**c**) The localisation of components of the motor complex was analysed using specific antibodies (MLC1, GAP40, GAP45) or co-expression of YFP-tagged constructs (*yfp-gap50*, *imc1-yfp*, *yfp-myoA*). MyoA_tail_ expression, as visualised using a specific antibody against the epitope tag Myc, resulted in a severe defect in parasite morphology, however glideosome components remained at the periphery of the mother cell. Scale bar 10 μm.

**Fig 2:**
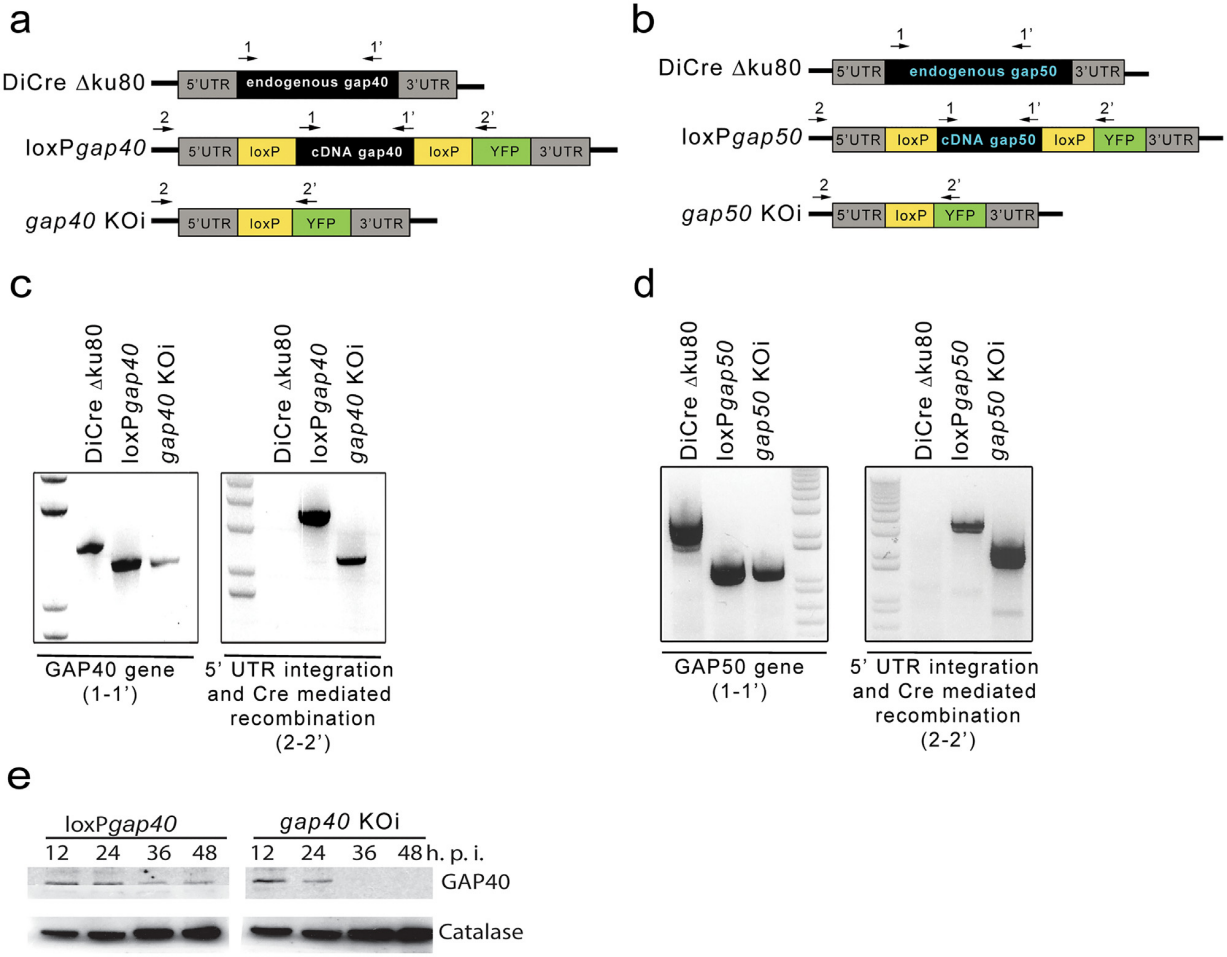
Strategy used and confirmation of integration of conditional deletion of *gap40* and *gap50*. Schematic indicating the strategy used to replace the endogenous *gap40* (**a**) or *gap50* (**b**) gene with the respective cDNA flanked by loxP sites. Arrows indicate location of oligonucleotides used for analytical PCRs. Integration PCRs demonstrating the correct integration of the *gap40* (**c**) or *gap50* (**d**) into the genome. Replacement of the endogenous gene with cDNA can be detected using primers (1–1’) while (2–2’) demonstrates correct 5’ integration (see Table 1) and Cre-mediated recombination. (**e**) 50 nM rapamycin was added to induce excision and parasites collected at indicated time points. Using a specific antibody, GAP40 levels are undetectable by 36 h post induction by western blot. Catalase was used as a loading control.

**Fig 3:**
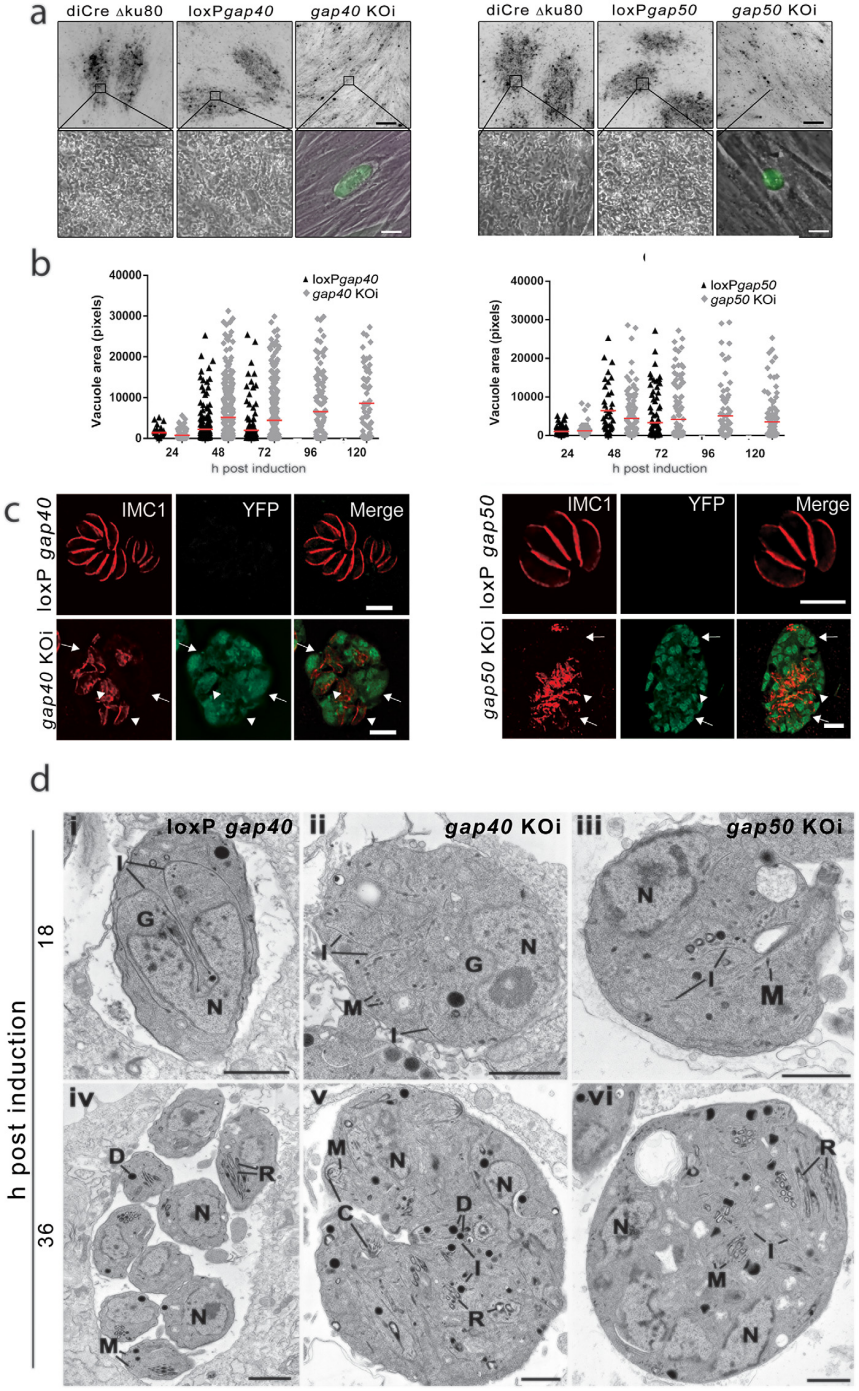
Conditional deletion of *gap40* and *gap50* leads to collapse of the IMC. (**a**) Growth assays of *gap40* and *gap50* KOi parasites. While control parasites showed normal growth behavior after 5 days of incubation, parasites lacking either *gap40* or *gap50* displayed no plaque formation on HFF monolayers. Scale bars represent 0.2 mm (upper panels) and 20 μm (lower panels). (**b**) The area of vacuoles at various time points post induction was quantified. In loxP*gap40* and loxP*gap50* parasites, vacuole size increased up to 48 h before reducing due to egress and reinvasion. Both *gap40* KOi and *gap50* KOi vacuoles behaved in a similar manner to the controls up to 48 h. However, no parasite egress was seen and vacuoles within host cells were maintained for at least 120 h post induction. Each point represents one vacuole (black for loxP and gray for KO) and results are representative of three independent experiments. Red line indicates mean vacuole area. (**c**) At 24 h post induction an antibody against IMC1 was used to visualise the IMC. Affected parasites lost peripheral staining and instead sheets of IMC1-positive structures were seen throughout affected vacuoles. Scale bar 10 μm (**d**) Ultrastructural appearance of WT (i, iv), *gap40* KOi (ii, v) and *gap50* KOi (iii, vi) parasites at 18 h (i, ii, iii) and 36 h (iv, v, vi) post induction. Scale bar 1 μm. (**i**) Longitudinal section through a parasite undergoing endoyogeny showing the conical shaped IMCs (I) of the two daughters partially enclosing the dividing nucleus (N). (**ii**) and (**iii**). Sections through the parasites showing the nucleus and areas of disorganised IMC (**iv**) Section through a parasitophorous vacuole showing a number of daughters forming a rosette. (**v**) and (**vi**) Sections through the parasite showing the cytoplasm containing multiple nuclei and area of apical formation consisting of the conoid and associated IMC, rhoptries and micronemes. The IMC appeared to be disorganised and does not form the conical structures associated with daughter formation. N—nucleus, I—IMC, C—conoid, R—rhoptry; M—microneme; D—dense granule G—Golgi body.

Previously, we demonstrated that overexpression of a dominant negative fragment of MyoA (ddMyc-MyoA_tail_) via the destabilisation domain (dd) system (as described in [22]) caused a defect in IMC biogenesis, in a similar manner to ablation of Rab11A or Rab11B function [19,20], suggesting a role of MyoA in IMC biogenesis. Detailed characterisation of this phenotype demonstrated that upon stabilisation of MyoA_tail_ using 1 μM Shield, the parasites displayed significantly aberrant morphology as revealed by electron microscopy (Fig 1a). Closer examination of major organelles revealed no significant alterations in the division of Golgi, apicoplast or in biosynthesis of the secretory organelles (S1 Fig). In contrast, the mitochondria appeared collapsed in Shield-1-treated parasites although it is not known if this is a primary effect of MyoA_tail_ expression or secondary to the IMC collapse (Fig 1b). Interestingly, despite the severe phenotype, components of the glideosome remained localised to the periphery of the parasite and the core components GAP45 and MyoA remained co-localised (Fig 1c). Previously it has been shown that knock out of MyoA does not have a similar effect on parasite morphology [20,21], suggesting that either other myosins are able to complement a possible role of MyoA during IMC formation, or, that overexpression of a fragment of MyoA has a deleterious effect on other key pathways within the cell. To test this we pulled down MyoA_tail_ and verified an interaction between MyoA_tail_ and GAP40 (S2 Fig), leading to the hypothesis that GAP40 could be involved in IMC biogenesis. In order to test this, we used the DiCre system as previously described [21] to conditionally delete *gap40* and *gap50* (Fig 2a). Replacement of the endogenous locus by the respective gene-swap vector [21] was confirmed using genomic PCR with indicated oligonucleotides (Table 1; Fig 2a and 2b) and both conditional knockouts demonstrated high excision efficiency upon induction of DiCre with rapamycin, resulting in an almost pure population of *gap40* and *gap50* null mutants (Fig 2c and 2d). Using a specific antibody we confirmed gradual loss of GAP40 protein over time. By 36 h GAP40 was undetectable by western blot (Fig 2e) and in affected vacuoles by IFA (S3 Fig). Unfortunately, we were unable to perform a similar analysis for GAP50 protein levels due to cross reactivity of GAP50 antibodies. Both *gap40* and *gap50* are essential for parasite survival as removal of either gene resulted in abrogation of parasite proliferation as seen in a growth assay (Fig 3a). Although parasites were unable to form plaques on a monolayer of HFF cells, large YFP-positive vacuoles could be observed after five days of incubation. In order to quantify the size of these vacuoles, induced parasites were fixed at different time points and vacuole area was determined. In control parasites (loxP*gap40* and loxP*gap50*), mean vacuole size increased between 24 and 48 h before decreasing at 72 h, due to egress and reinvasion (Fig 3b). After induction, both *gap40* KOi and *gap50* KOi parasites displayed similar behaviour compared to the controls for the first 48 h. However, at later time points, vacuole size was maintained for the duration of the experiment and no evidence of egress and reinvasion was observed. Together these results indicate that upon deletion of *gap40* or *gap50*, parasites are capable of expansion but are unable to lyse the host cell.

**Table 1:** List of oligonucleotides used in this study.

Name	Sequence (5’– 3’)
*gap50* cDNA FW	GGGTTAATTAATTATTTCATGTAGCGAGAGAGACCGTTC
*gap50* cDNA RV	CCCAATTGGACAAAATGGCAGGCGCCCCCGTCGCGG
5’ UTR *gap50* RV	CGGAATTCTATAACTTCGTATAATGTATGCTATACGAAGTTATAGTTTGGAG TTGGCCGAGAGCAGGAAAACGGTTCCCGAAA
5’ UTR *gap50* FW	GGGCCGGCTGCTTGA TGAGTCAGCGCA TGT A TGTGTTTTG
5’ UTR *gap40* FW	CGGGCCGGCCAGATAGCCTGTCCACCCTCAGTACGC
5’ UTR *gap40* RV	CGAGATCTTATAACTTCGTATAATGTATGCTATACGAAGTTATCCAAACGTCT GAAAAAGGCAGAGGAACTGTGCGAACGCTGG
*gap40* cDNA FW	CCGCAATTGAGATCTATGTCGACTCTTCAGGACATTCGCTTG
*gap40* cDNA RV	GGGTTAATTAATGCATTAGCTCGAATGGGCTTCGTCGTCAC
5’ UTR *gap50* 2 FW	CCCTGCGTAGCAAAAGTCGGACTC
3’ UTR *gap50* 2 FW	CGAGCATCCGACATCTACCTGTACTGACC
3’ UTR *gap40* 2 RV	CCGCTGGAGAAAACCCAAGTACATTGC
5’ UTR *gap40* 2 RV	GCAGGCTCCATCCAAACCCAAAGTC

### Deletion of *gap40* or *gap50* results in a significant defect in inner membrane complex (IMC) morphology and the parasites ultrastructure

As *gap40* KOi and *gap50* KOi parasites were unable to complete the lytic lifecycle, we investigated intracellular development of parasites in detail. Parasites were fixed at 24 h post induction and the morphology of the parasites visualised using an antibody against the alveolin IMC1. While the morphology of loxP*gap40* and loxP*gap50* parasites appeared unchanged, deletion of either *gap40* or *gap50* (Fig 3c) resulted in a dramatic redistribution of the parasite IMC. Peripheral IMC1 staining appeared completely abolished (arrow) and disorganised staining of IMC can be seen within vacuoles (arrowheads), potentially representing sheets of collapsed IMC (Fig 3c). Interestingly these defects in IMC formation at 24 h post induction occur when parasites still contained significant levels of GAP40 (Fig 2e), suggesting that *de novo* production of GAP40 is required by the parasite.

In order to further examine the effects of deletion of *gap40* or *gap50*, the ultrastructure of induced parasites was examined using transmission electron microscopy. At 18 h post induction, the WT parasites showed evidence of undergoing endodyogeny with the formation of the conical shaped IMCs of the two daughters within the wild type cell cytoplasm (Fig 3di). In contrast, the *gap40* KOi and *gap50* KOi parasites appeared spherical and with little obvious polarization (Fig 3dii, 3diii, 3dv and 3dvi) although the maternal cell conoid could be recognised in some sections (Fig 2diii). Within the cytoplasm it was possible to identify individual plates of the IMC, but these were disorganized and did not appear to be coordinated to form the conical-like structures associated with daughter formation in the WT (Fig 3dii and 3diii).

By 36 hours post excision, many of the host cells contained large parasitophorous vacuoles (PV) with multiple daughters consistent with repeated endodyogeny (Fig 3div). Both mutant parasites showed significantly different cytoplasmic appearances to those of the WT at the same time point (Fig 3div, 3dv and 3dvi). Many of the spherical shaped parasites showed an increase in size compared to 18 h post induction (Fig 3dv and 3dvi). At this stage, the majority of parasites had cytoplasm containing multiple nuclei and multiple examples of daughter formation, recognised through the appearance of the conoid with associated IMC and apical organelles (rhoptries, micronemes and dense granules) (Fig 3dv and 3dvi). However, the sheets of IMC associated with daughter cells appeared disorganized, although some had associated microtubules, and did not form the organized conical structures associated with daughter cell formation. In addition there was no obvious association of the cell plasma membrane with the IMC to form the pellicle (Fig 3dv and 3dvi). In summary, the *gap40* KOi and *gap50* KOi parasites appeared to be unable to form organised daughter cells, although all components were present.

### Deletion of gap40 or gap50 results in alteration in the localisation of glideosome components

**Fig 4:**
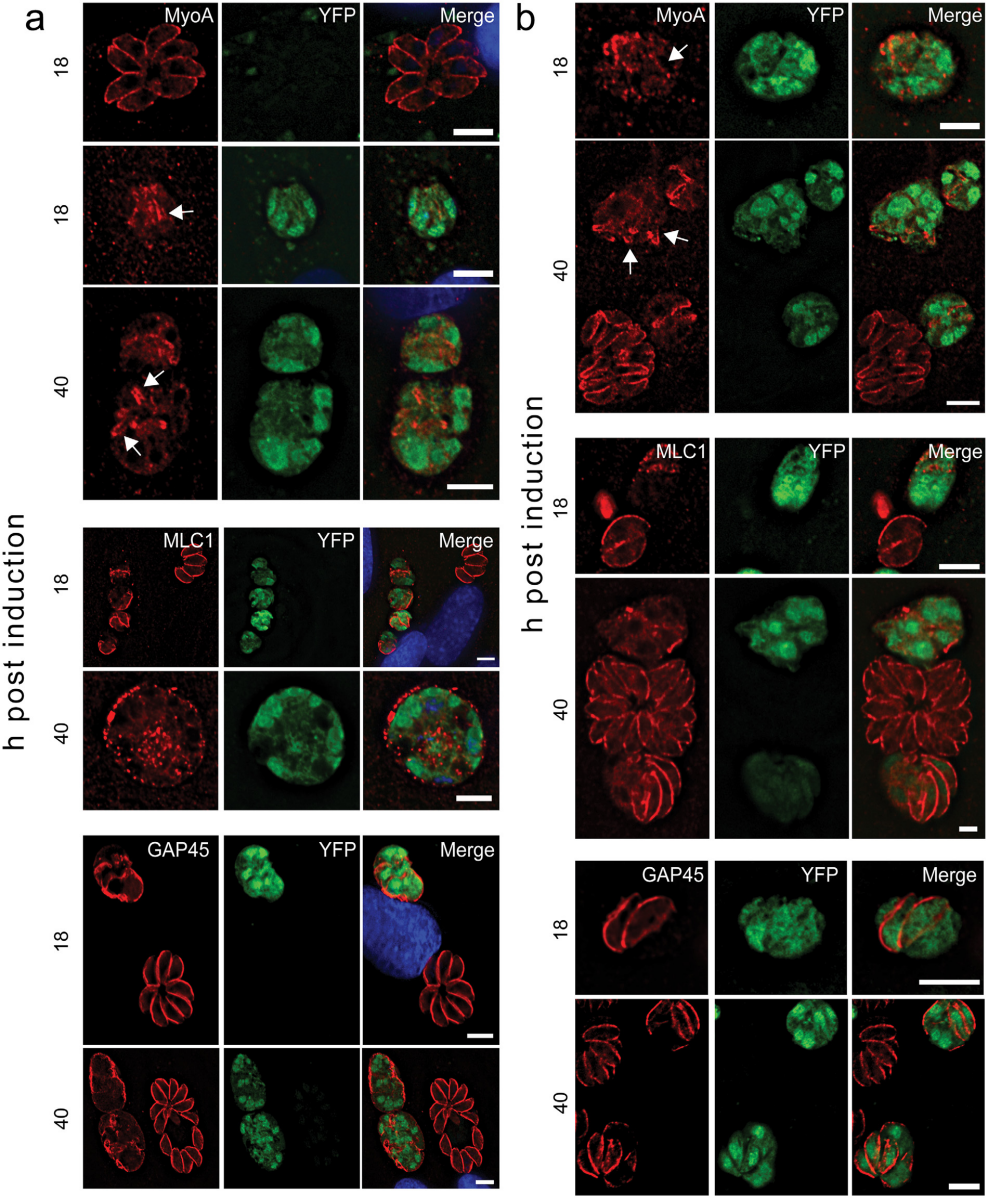
Conditional deletion of *gap40* or *gap50* results in altered localisation of components of the glideosome. (**a**) Deletion of *gap40* leads to loss of MyoA staining from the periphery of the vacuole at 18 and 40 h post induction although some MyoA-positive structures were visible within the vacuole (arrows). Using a specific antibody, MLC1 localisation was also significantly affected at both time points tested. GAP45 was still seen at the periphery of vacuoles however abnormal structures were visible and there was no delineation between parasites in the same vacuole. (**b**) Similar results were observed upon deletion of *gap50*, with a loss of peripheral MyoA staining and abnormal morphology using MLC1 and GAP45 antibodies. Scale bar 10 μm.

Next we examined the localisation of other components of the glideosome. In both *gap40* KOi and *gap50* KOi parasites, MyoA localisation was abolished with small structures, potentially corresponding to sheets of IMC remnants, observed within the parasites (arrows) (Fig 4a and 4b). The localisation of the MyoA light chain, MLC1, was also altered in the absence of both *gap40* and *gap50*, either becoming more vesicular with a concentration around the periphery of the vacuole or by mislocalising entirely. In contrast, GAP45 appeared to remain associated with the peripheral membrane of the vacuole (Fig 4a and 4b), possibly due to the acylation and insertion of the N-terminus of the protein into the plasma membrane [16]. The alteration of localisation of MyoA/MLC1 is believed to be due to the disruption and fragmentation of the IMC rather than dissociation of the glideosome complex. This hypothesis is supported by the partial co-localisation of MLC1 with IMC1 in the *gap40* KOi, suggesting that MLC1 is still associated with the fragmented IMC while excess MLC1 is cytosolic (S4 Fig). This is in contrast to the knockout of GAP45 which did not affect the structure of the IMC but resulted in cytoplasmic localisation of MyoA/MLC1 [11]. These data, in combination with previous data [14], suggest that GAP40 or GAP50 may be dispensable in the anchoring of the glideosome complex to the IMC but do have essential roles in the stabilisation and formation of the inner membrane complex itself.

As the phenotype of *gap40* KOi and *gap50* KOi parasites appeared identical by both immunofluorescence and transmission electron microscopy, it was decided to focus on the characterisation of the *gap40* KOi in order to analyse the dynamics of IMC formation in more detail.

### GAP40 is not required for division of the nucleus, apicoplast or Golgi

*T*. *gondii* replicates in an orchestrated, highly regulated process as recently described [23]. Briefly, the centrioles, Golgi and apicoplast are first observed to duplicate, followed by nuclear division [24,25]. Daughter cell scaffold construction is initiated and the IMC elongates in a microtubule-dependent manner [26]. Once the daughter scaffolds have elongated, the mitochondrion is packaged into the daughter cells, secretory organelles are made *de novo* and the daughter cells separate, leaving behind a residual body [24]. In order to determine at what point in the cell cycle *gap40* was required, the morphology of major organelles during cell division was analysed. At 24 h post induction of excision of *gap40*, no significant effect on nuclear replication was observed (S5 Fig).

Furthermore, deletion of *gap40* did not affect Golgi replication or segregation (S5 Fig). Although vacuolar organisation was significantly affected due to the lack of IMC, apicoplast replication and segregation appeared normal, as the ratio of apicoplasts to nuclei remained constant (S5 Fig), confirming previous reports that apicoplast replication is tightly linked to nuclear division via association with centrosomes, but not IMC elongation or maturation *per se* [25]. Both rhoptries and micronemes could generally be observed in affected parasites (S5 Fig), confirming previous results that biogenesis of secretory organelles is not functionally linked to IMC formation [19,20]. Interestingly, although the mitochondrion was seen to expand, mitochondrial segregation appeared abnormal (S5 Fig). At 40 h post induction, this resulted in a large interconnected mitochondria (S5f Fig), suggesting that a functional IMC is likely required for mitochondrial cleavage and segregation.

### Initiation of daughter cell formation is not dependent on *gap40*

**Fig 5:**
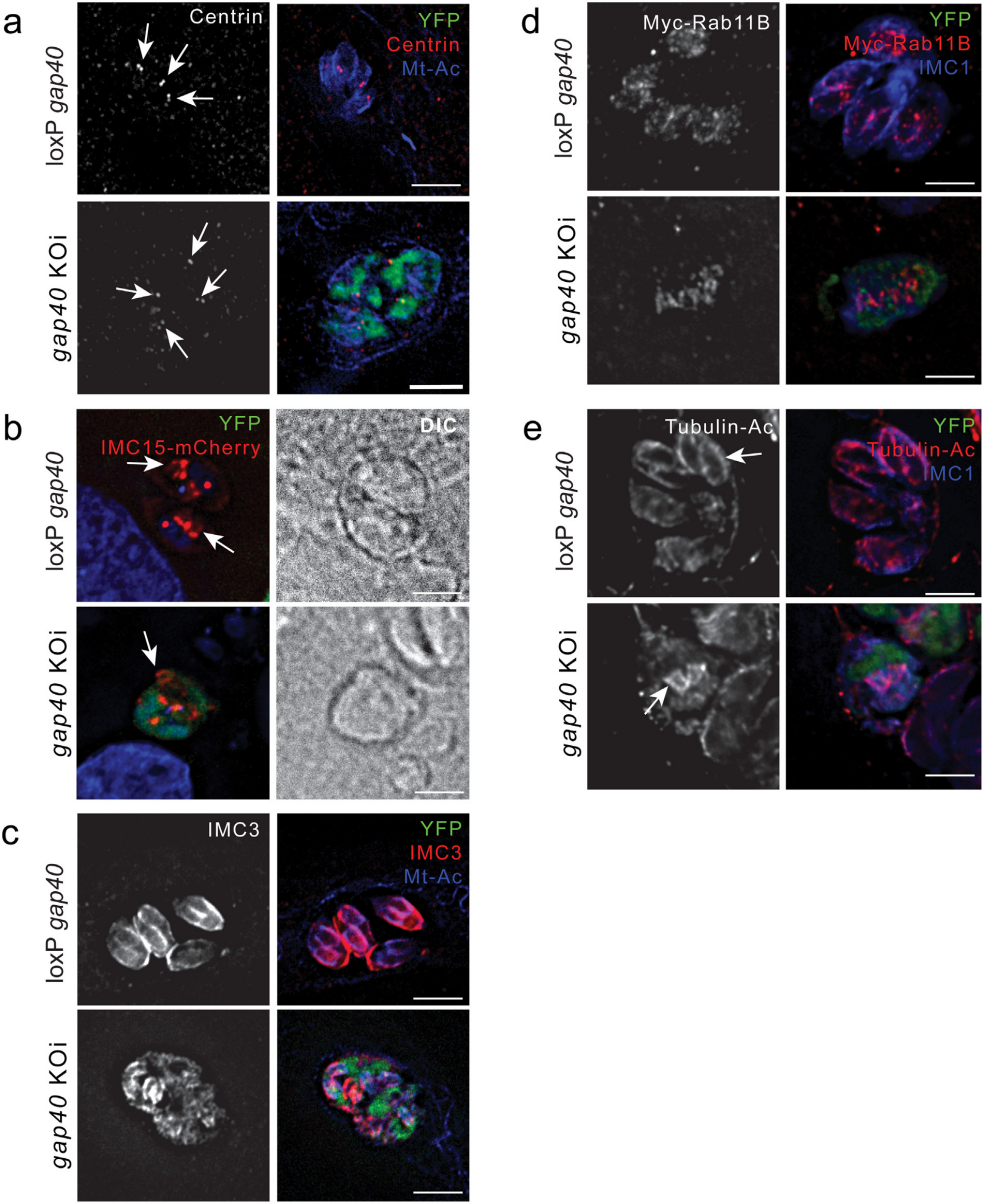
Early markers of daughter cell formation are recruited in *gap40* KOi parasites. Parasites were induced and fixed at 24 h post addition of rapamycin. (**a**) At this time point, multiple structures labelling with a specific anti-centrin antibody can be seen in *gap40* KOi vacuoles (arrows). (**b**) Transient expression of IMC15-mCherry demonstrates structures resembling daughter cell buds (arrows). This is confirmed by the recruitment of IMC3 using a specific antibody (**c**), transiently expressed Myc-Rab11B (**d**) and an antibody against acetylated tubulin (**e**) to newly forming daughter cells. Scale bar 5 μm.

Deletion of *gap40* resulted in a severe defect in the morphology of the parasites however did not appear to significantly affect organelle biogenesis. We therefore hypothesised that *gap40* was required for daughter cell scaffold formation. In order to define the function of *gap40* during IMC biogenesis, markers for early cell division, such as centrin [25], IMC15 [5], IMC3 [5,27] or Rab11B [19] were employed. This analysis demonstrated that *gap40* KOi parasites duplicated their centrosome although individual parasites could not be distinguished (Fig 5a, arrows). The IMC proteins, or alveolins, are a family of intermediate filament-like proteins that are sequentially assembled in forming daughter cells [5]. The first of these recruited to daughter cells, IMC15-mCherry, was expressed in *gap40* KOi parasites and shapes reminiscent of daughter cell formation were observed (arrows) (Fig 5b). The early daughter cell marker IMC3 was also observed in similar structures (Fig 5c), suggesting that early alveolin markers of the IMC are recruited to nascent daughter cells despite the significant IMC disruption in the knock out parasites. The small GTPase Rab11B is essential for IMC biogenesis in developing daughter cells [19]. In *gap40* KOi parasites, recruitment of Rab11B fused to a Myc tag and FKBP destabilisation domain was observed at structures resembling daughter cells (Fig 5d). *Toxoplasma* microtubules are heavily acetylated [28,29] and we have found that antibodies against acetylated tubulin are very effective at visualising daughter cell buds. Using an antibody for acetylated tubulin formation of early daughter cell scaffolds could be visualised in both the KO and loxP parasites (arrows, Fig 5e). Together these data strongly suggests that initial daughter cell development can occur in parasites devoid of GAP40. The presence of these markers, in combination with the apical complexes visualised by electron microscopy (Fig 3d), demonstrates that daughter cell formation occurs in the *gap40* KOi parasites. However, in all cases the IMC of daughter cells appears abnormal and no full assembly of the IMC can be observed, strongly indicating that while GAP40 is not required for initiation of daughter cell formation, it fulfils a crucial role in ensuring the stability of the *de novo* formed IMC of daughter cells.

### *gap40* KOi parasites begin to collapse soon after initiation of replication

**Fig 6:**
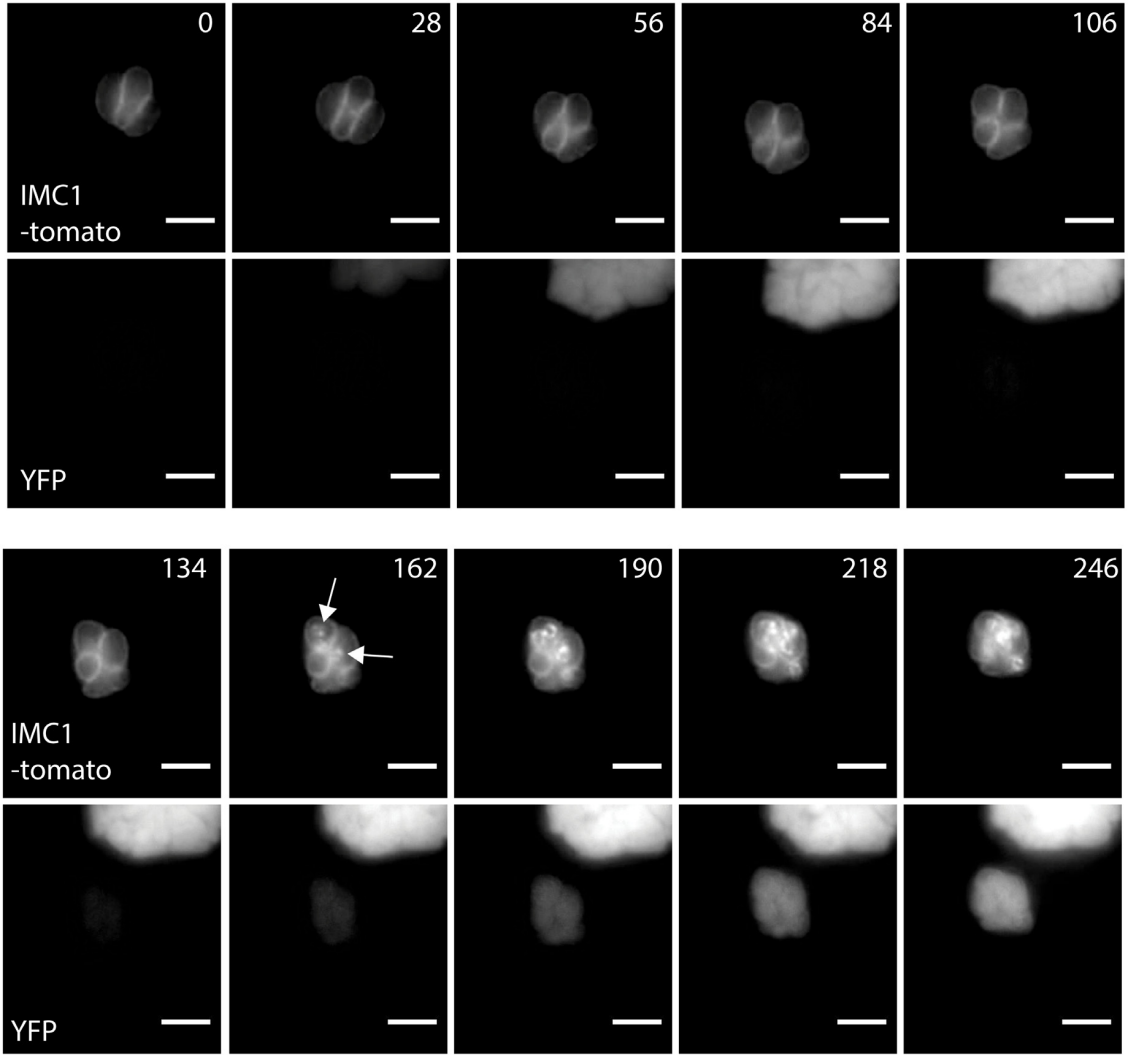
*gap40* KOi parasites collapse after initiation of the cell cycle. Parasites were transiently transfected with *imc1-tomato*, allowed to express overnight before the addition of 50 nM rapamycin to induce excision of the gene. 4 hours post excision, imaging was initiated and images taken every 7 minutes, every second frame is displayed. The IMC1-expressing vacuole initiated endodyogeny at 162 min after imaging was initiated as indicated by the formation of daughter cell buds (arrows) and at the same point YFP expression was observed. Soon after the initiation of endodyogeny, the vacuole was observed to lose organization. Scale bar 5 μm.

In order to follow the intracellular development of *gap40* KOi parasites in real time, we expressed IMC1-tomato to allow visualisation of IMC biogenesis in replicating parasites. 4 h post excision of *gap40*, cells were imaged every 7 minutes for around 4 h (Fig 6, S1 Movie, loxP*gap40* parasite replication is shown in S2 Movie). In the image shown, daughter cell buds were first seen at 162 min after start of image capture (arrows) and appeared to elongate normally until approximately 218 min where disorganisation of the IMC became evident. By the end of imaging, the parasites had acquired the characteristic collapsed morphology. Initiation of daughter cell budding coincided with YFP signal becoming detectable, suggesting tight transcriptional control of *gap40*. Indeed, both *gap40* and *gap50* are transcribed in a cell cycle-dependent manner with initiation of transcription during G1 phase and peaking in S1/M phase [30].

**Fig 7:**
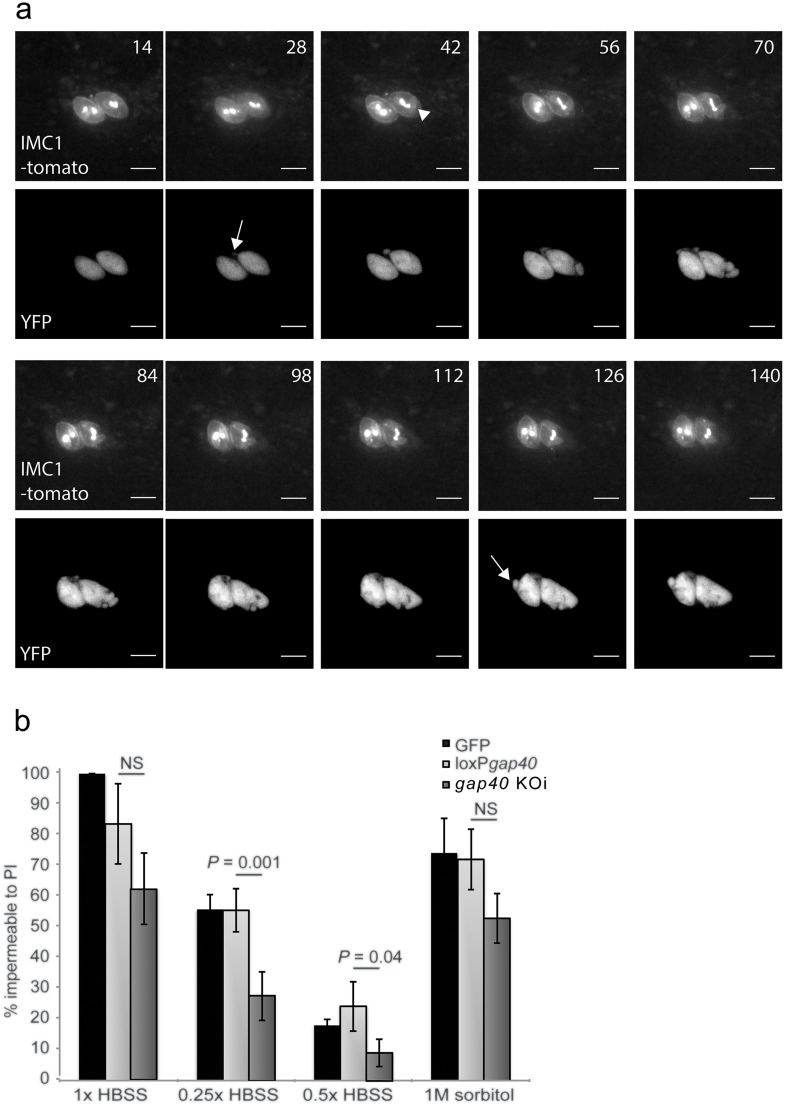
*gap40* KOi parasites show dissociation between the plasma membrane and the IMC and show reduced ability to resist osmotic shock. (**a**) *gap40* KOi parasites were transiently transfected with *imc1-tomato*, incubated overnight to allow protein expression then excision was induced. 4 h after the addition of rapamycin, imaging was initiated and images captured every 7 mins, every second image is displayed. YFP signal was observed to appear outwith the IMC boundaries (arrows) followed by loss of the parasite integrity by 42 min (arrowhead). Scale bar 5 μm. (**b**) At 24 h post induction, parasites were mechanically lysed and treated with either HBSS, HBSS diluted with distilled H_2_O (hypoosmotic) or 1 M sorbitol/HBSS (hyperosmotic) as indicated for 5 mins. The percentage of parasites that remained impermeable to PI was then scored and normalized to untreated GFP parasites. *P* values are shown between the loxP*gap40* and *gap40* KOi strains. NS—not significant. Results mean of three independent experiments ± standard deviation.

During the same time lapse analysis we observed that in several cases YFP signal appeared outside the boundaries of the IMC but within the parasite plasma membrane and the parasitophorous vacuole (PV) during the collapse of *gap40* KOi (Fig 7a, arrows). This indicates at least partial dissociation between the plasma membrane and the IMC (Fig 7a, S3 and S4 Movies). Next, extracellular parasites were subjected to different osmotic stress conditions and *gap40* KOi parasites demonstrated a lower survival rate compared to control parasites in increasing concentrations of water (Fig 7b). These data suggest that GAP40 has important roles in maintaining the structural integrity of the parasite and that *de novo* GAP40 production is required to maintain the parasite’s stability.

### GAP40 is localised to foci along the IMC and is distributed in a non-random fashion

**Fig 8:**
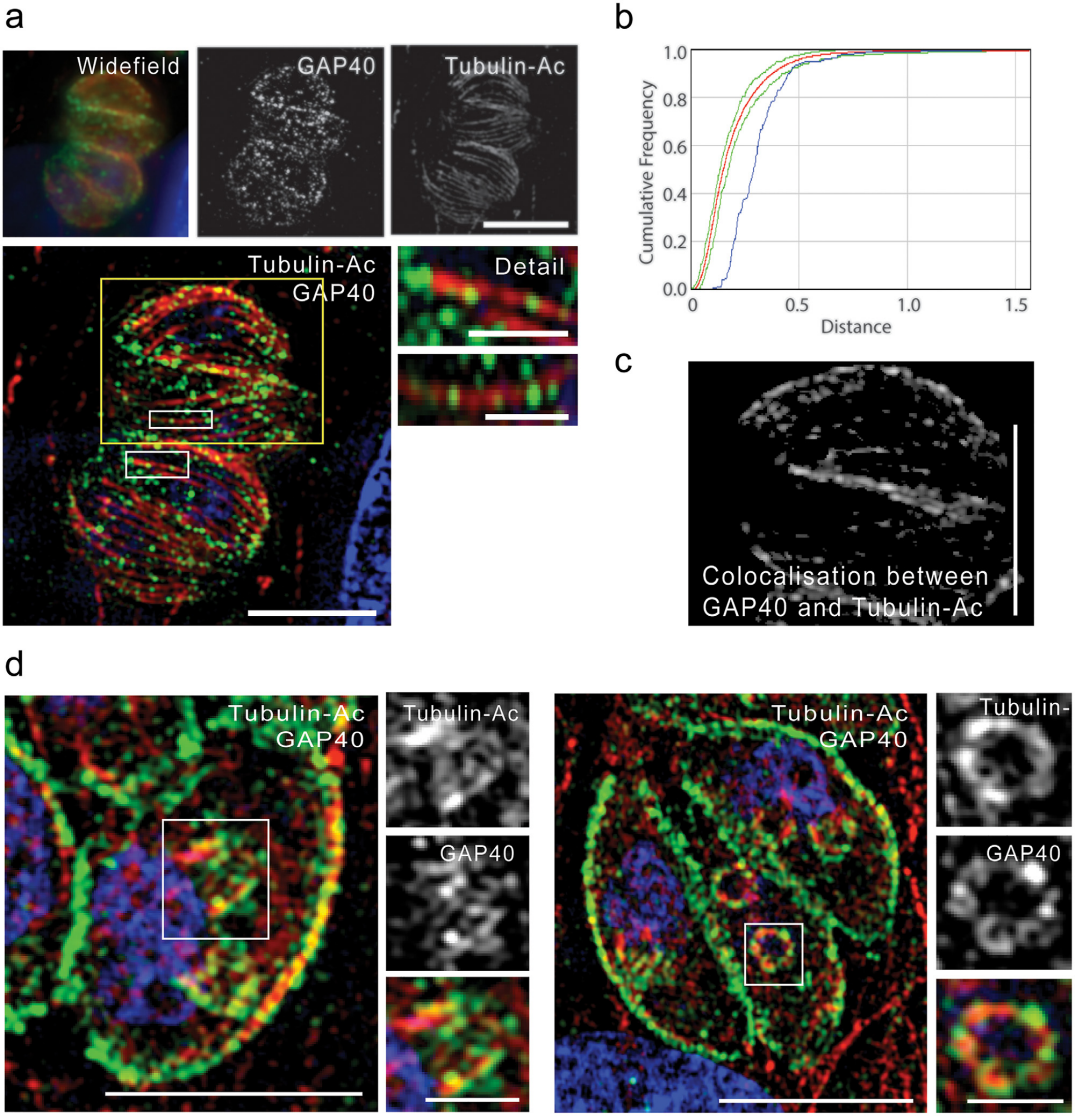
SR-SIM reveals novel GAP40 localisation. Parasites were fixed and stained as indicated with specific antibodies against GAP40 (green) and acetylated tubulin (red) (**a**) SR-SIM microscopy allows individual microtubules to be clearly distinguished compared to conventional wide field microscopy. GAP40 localisation was observed as foci of staining which appeared to correlate with the path of subpellicular microtubules. Scale bar 5 μm, detail images scale bar 1 μm. (**b**) Statistical analysis of GAP40 foci distribution in a single cell. Foci demonstrate a more regular distribution (blue line) than predicated by chance (red line, 95% confidence levels indicated by green lines). Graph representative of at least 20 separate cells from two independent experiments. (**c**) Co-localisation between a subset of GAP40 foci and Tubulin-Ac was observed, image shows only co-localizing pixels and is a projection of the entire Z-stack. Scale bar 1 μm. (**d**) GAP40 was also closely associated with microtubules in very early stages of daughter cell scaffold construction. Scale bar 5 μm, detail images scale bar 1 μm.

GAP40 has previously been localised to the alveoli of the IMC [16]. However, recent studies have demonstrated that there are multiple, distinct domains within the alveoli. Localisation of ISP proteins has demonstrated three sub-domains of the alveoli [31] while SIP, CBAP and ISC3 have been hypothesised to delineate the joining plates of the alveoli [32–34]. In order to analyse the localisation of GAP40 in more detail, we used super-resolution structured illumination microscopy (SR-SIM). We observed for the first time that GAP40 is seen in small foci at the IMC (Fig 8a). Statistical analysis of these foci (using the method previously described [35,36]) suggested that these foci were distributed more regularly (Fig 8b, blue line) than chance would predict (red line, green lines indicate 95% confidence limits). Using an antibody against acetylated tubulin, we saw that a proportion of GAP40 foci appeared to be distributed along the path of subpellicular microtubules in mature parasites (Fig 8a, detail). Indeed, limited co-localisation between tubulin and GAP40 was observed despite the expected lack of direct interaction between these proteins (Fig 8c). Localisation along the path of microtubules was also seen in very early daughter cell buds (Fig 8d). In contrast, both GAP45 (S6 Fig) and MLC1 (S6 Fig) demonstrated a smooth localisation along the surface of mature parasites which did not appear to follow the path of microtubules. Statistical analysis showed that these proteins were distributed randomly over the surface of the IMC (S6 Fig)

### Disruption of members of the GAPM family of proteins leads to collapse of the IMC

**Fig 9:**
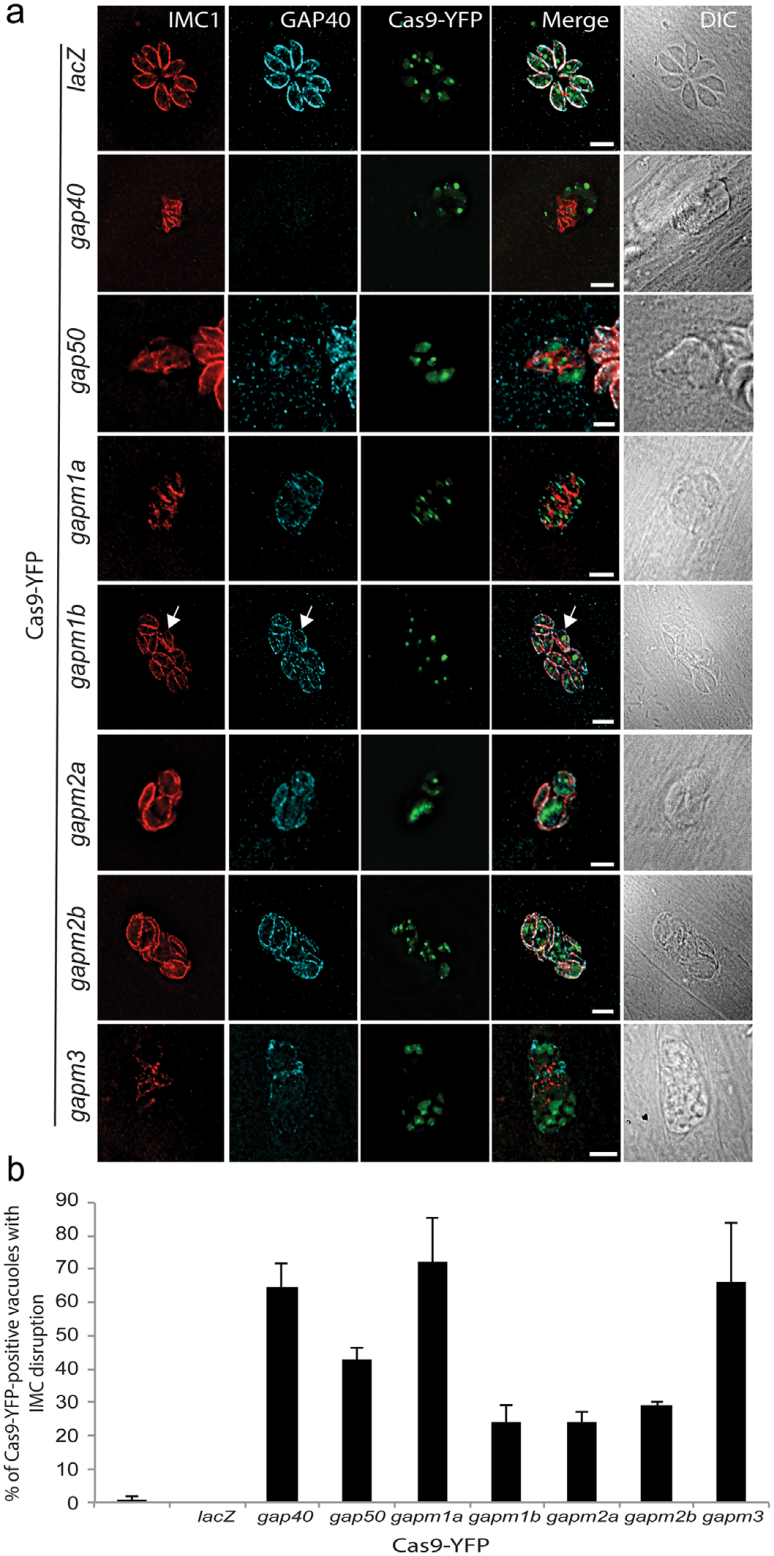
Disruption of *gapm* genes using CRISPR-Cas9 leads to collapse of the IMC Parasites were co-transfected with Cas9-GFP and gRNA targeted to indicated genes. (**a**) At 48 h post transfection, parasites were fixed and visualized using anti-IMC1 and anti-GAP40 antibodies. Parasites transfected with sgRNA targeting *gap40* showed collapse of the IMC and an absence of GAP40 staining, while disruption of *gap50* also resulted in structural collapse, confirming the utility of this technique. Disruption of *gapm* genes resulted in morphological abnormalities to varying degrees, suggesting that *gapm1a* and *gapm3* are essential while disruption of *gapm1b* had a more subtle effect on parasite morphology. Scale bar 5 μm. (**b**). Quantification of percentage of vacuoles positive for Cas9-GFP that showed disruption of the IMC at 48 h post transfection. While between 60–70% of GFP-positive vacuoles showed disruption to the IMC upon co-transfection with Cas9-GFP and *gap40*, *gapm1a* and *gapm3*, less than 30% of vacuoles co-transfected with *gapm1b*, *gapm2a* and *gapm2b* demonstrated severe phenotypes. Results average of three independent experiments, performed in duplicate ± standard deviation.

The GAPM proteins are multiple membrane spanning proteins conserved between the apicomplexa. These proteins have previously been localized to the IMC in both *T*. *gondii* and *P*. *falciparum* schizonts and members of this protein family have been shown to pull-down components of both the glideosome as well as alveolins [37]. However, the function of these proteins remains unknown. We hypothesized that given the structural roles of GAP40 and GAP50; the GAPM proteins may also have a role in stabilizing the nascent IMC. The five GAPM members found in *T*. *gondii* were disrupted using the CRISPR-Cas9 gene disruption system recently validated for use in *T*. *gondii* [38,39]. Parasites were transiently co-transfected with a plasmid expressing the Cas9 endonuclease fused to GFP and a nuclear localization sequence in combination with small guide RNA (sgRNA) targeting each of the five members of the GAPM family (Table 2). Transfection with a sgRNA targeting the *lacZ* gene had no morphological effects at 48 h post transfection, while parasites co-transfected with the *gap40* or *gap50* sgRNA demonstrated collapse of the IMC, in an identical manner to that achieved with the conditional DiCre KO system (Fig 9a). Parasites transfected with sgRNA targeted to *gapm1a* and *gapm3* demonstrated a severe loss of normal morphology including collapse of the IMC in a very similar manner to the *gap40* KOi and dissociation between the sub-pellicular marker IMC1 and the alveolar marker GAP40. This dissociation was not seen upon disruption of *gapm2a* and *gapm2b* which instead resulted in a less severe IMC phenotype in affected vacuoles. Upon expression of *gapm1b s*gRNA, the IMC was often relatively normal however areas were seen at the periphery of parasites that appeared to have no or little IMC1 (Fig 9a, arrow). Quantification of affected vacuoles revealed between 60–70% of GFP-positive vacuoles demonstrated IMC disruption upon transfection of *gap40*, *gapm1a* and *gapm3* sgRNAs, around 40% of vacuoles transfected with *gap50* sgRNA and only around 25% of vacuoles expressing *gapm1b*, *gapm2a* and *gapm2b* sgRNAs (Fig 9b). The similarities in phenotype between disruption of *gap40* and the *gapm* genes, combined with previous data strongly suggests a direct interaction between these proteins [37] and a functional link between the glideosome-anchors GAP40 and GAP50 and the GAPM family of proteins in the IMC.

**Table 2:** List of gRNAs used in this study.

**Gene name**	**Gene ID**	**sgRNA recognition sequence**	**PAM**
*lacZ*	N/A	GATGAAAGCTGGCTACAGGA	AGG
*gap40*	TGGT1_249850	TGGAGTACAGCACTGCCCAT	GGG
*gap50*	TGGT1_219320	CGTGGGGGTGGCAAGGAAAA	CGG
*gapm1a*	TGGT1_202500	TGCTGCTGCGGAGCAGGCTC	AGG
*gapm1b*	TGGT1_202510	GGAAGCCAAGTCTCAAGTTG	TAG
*gapm2a*	TGGT1_219270	TCGTCAGGGTACCGGCTACA	TGG
*gapm2b*	TGGT1_206690	CCTGAAGACACGATCCGGTT	CGG
*gapm3*	TGGT1_271970	TACTGGACCTTTGGAGGATC	AGG

## Discussion

Recently, a number of studies have established that the makeup of the IMC is highly dynamic throughout the asexual lifecycle and that a number of proteins localise to distinct domains [32–34,40]. However, only a few IMC-resident proteins have thus far been investigated in detail and our knowledge of essential factors required for biogenesis and integrity of the IMC remains limited.

Apart from its structural role during daughter cell assembly, the outer leaflet of the IMC serves as anchor for the glideosome, which is important for gliding motility and host cell invasion [13,16]. Previously, we have reported that overexpression of MyoA_tail_ leads to a strong replication defect and we speculated that the glideosome may have a role during IMC biogenesis [20]. However, conditional null mutants of the key components *act1*, *myoA*, *mlc1* and *gap45* [11,16,21,41] have no defect in IMC biogenesis, suggesting that actin-myosin motors are not directly involved in the vesicular transport to the IMC. Instead, it appears that MyoA_tail_ does not act as a ‘true’ dominant negative mutant, but disrupts the function of other components of the MyoA-motor complex in a concentration-dependent manner. Indeed co-IP analysis demonstrates that MyoA_tail_ interacts with GAP40 and we establish here that deletion of *gap40* and *gap50* results in a similar phenotype to MyoA_tail_ overexpression. GAP40 and GAP50 are both transmembrane proteins which have been hypothesised to anchor and immobilise the glideosome in the IMC [13,16]. The detailed analysis of conditional null mutants for *gap40* and *gap50* demonstrates for the first time that components of the gliding machinery have distinct and dual functions during the asexual lifecycle of the parasite. In contrast to a mutant for Rab11B, where IMC biogenesis appears to be abolished [19], early daughter cell development can be readily observed upon depletion of GAP40 or GAP50. Therefore, we conclude GAP40 and GAP50 are not required for initiation of daughter cell formation or vesicular transport to the IMC *per se*, but rather have a role in IMC biogenesis and stabilisation. Interestingly, the results we present here suggest that neither GAP40 nor GAP50 are individually required for the localisation of the glideosome components MyoA, MLC1 and GAP45 to the inner membrane complex although it remains to be established if this reduced complex is functional. Future work will be required to assess if the glideosome is able to form and localise to the IMC in the absence of both putative anchor proteins. It is possible that other, currently unknown, proteins also have a role in anchoring the motor complex to the IMC.

Time-lapse analysis confirms that GAP40-depleted parasites initiate formation of daughter cell IMC, however both maternal and daughter cells become intrinsically unstable in the absence of GAP40 and start to lose organisation and integrity, leading to dissociation of the parasite IMC and plasma membrane and collapse of the vacuole. This instability may be caused by the recycling of maternal GAP40 to the daughters as was previously described [15] as it is seen soon after excision of the gene when high levels of protein still remains.

Interestingly, using super-resolution structured illumination microscopy (SR-SIM) we found that GAP40 has a punctuate localisation. This is in contrast to the localisation observed for other glideosome components, GAP45 and MLC1. The significance of this result is unknown, however early studies establishing the 1:1 stoichiometry of the glideosome were performed before the existence of GAP40 was established. One possible explanation is that GAP40 is only associated with a subset of complexes, with others perhaps being anchored by GAP50 or other, currently unknown, proteins. The distribution of GAP40 in the IMC is intriguing. Here we show that GAP40 foci are arranged in a regular fashion and we suggest that microtubules may have a role in the arrangement of foci in the IMC, however future studies will be required to further analyse this localisation and its functional significance.

Previously it has been shown that GAPM proteins could pull-down the glideosome complex [37]. Using the CRISPR-Cas9 system, we present the first evidence that GAPM1a and GAPM3 are likely essential for normal parasite development. Interestingly, in affected vacuoles there appears to be dissociation between the alveolar marker IMC1 and the integral IMC protein GAP40, which would agree with the predicted role of these proteins as a link between the sub-pelliclular network and the alveoli of the IMC. The role of the other members of the GAPM family is less clear with a proportion of affected vacuoles displayed IMC defects of varying degrees, suggesting that some members of this family may have some redundancy. However, in order to fully characterise the role (or roles) of this protein family, either clean or conditional knockouts are required.

In summary, we demonstrate that the glideosome proteins GAP40, GAP50 and members of the GAPM family have a crucial role in IMC biogenesis and stability during cell division, providing the first functional evidence of a role beyond parasite motility for components of glideosome.

## Materials and Methods

### *T*. *gondii* cultivation and transfection

*T*. *gondii* parasites (RH *Δhxgprt* unless otherwise indicated) were grown on human foreskin fibroblasts (HFF-1; purchased from ATCC SCRC-1041), transfected and selected as previously described [22,42]. Plaque assays were performed as previously described [18].

### Generation of plasmids and parasite lines

The *loxPgap40loxP—YFP—HX* and *loxPgap50loxP—YFP—HX* constructs were generated as described previously with minor alterations [11]. The *gap40* (TGGT1_249850) *and gap50* ORFs (TGGT1_219320) as well as the 5’ and 3’ UTRs were amplified from cDNA or gDNA using the oligonucleotides described in Table 1. All fragments were cloned into the parental vector *p5RT70loxPKillerRedloxPYFP—HX*.

The ddMyc-MyoA_tail_ line was generated as described previously [20]. To generate the conditional *gap40* and *gap50* KO strains, 60 μg of the plasmids *loxPgap40loxPYFP-HX* and *loxPgap50loxPYFP-HX* were transfected into a novel DiCre Δku80 strain (Pieperhoff *et al*., in preparation). The resulting loxP*gap40* and loxP*gap50* strains bear only the floxed copy of *gap40* or *gap50*, allowing excision upon addition of rapamycin (50 nM for 4 hours) to generate the *gap40* and *gap50* KOi mutant parasites (*diCre Δku80/gap40* ^−^, *diCre Δku80/gap50* ^−^) referred to here as *gap40* KOi and *gap50* KOi respectively). Verification of 5′ UTR integration in the loxP*gap40* strain and excision of *gap40* was confirmed by PCR using the oligo pair *gap40* 5′UTR fw (1) and YFP rv (1′). Correct integration into 3′ locus was analysed using the primers HX fw2 (2) and GAP40 3UTR rv (2′). Verification the loxP*gap50* strain proceeded in a similar manner, 5′ UTR integration and excision of *gap50* was confirmed by PCR using the oligo pair *gap50* 5′UTR fw (1) and YFP rv (1′). Correct integration into 3′ locus was analysed using the primers HX fw2 (2) and GAP40 3UTR rv (2′).

### Immunofluorescence

For immunofluorescence analysis, HFF cells grown on coverslips were inoculated with *T*. *gondii* parasites in absence or presence of 1 μM Shld-1 or 50 μM rapamycin for the time indicated in the text. Cells were fixed with 4% w/v paraformaldehyde in phosphate buffered saline (PBS) for 20 min at room temperature. Fixed cells were permeabilized and blocked using 0.2% Triton-X100, 2% bovine serum albumen (BSA) in PBS and stained accordingly. Primary antibodies used were mouse anti-Myc (Sigma, M-4439), rabbit anti-GAP40, rabbit anti-MLC1 and rabbit anti-catalase (kind gifts from Dominique Soldati), rabbit anti-GAP45 and rabbit anti-IMC1 (gifts from Con Beckers), mouse anti-Ty, rabbit anti-FKBP (Thermo Scientific, MA1-91878), rabbit anti-MyoA (a gift from Gary Ward), mouse anti-HSP60 and rat anti-IMC3 (gifts from Boris Striepen) mouse anti-centrin (Millipore, 04–1624), and mouse anti-acetylated tubulin (Sigma, T6793). Secondary antibodies used were goat anti-mouse, goat anti-rabbit or goat anti-rat AlexaFluor 350, AlexaFluor 488, AlexaFluor 594 or AlexaFluor 633 conjugated antibodies as required (Life Technologies).

### Microscopy and spatial distribution analysis

Widefield images were acquired in z—stacks of 2 μm increments and were collected using a UPLSAPO 100× oil (1.40NA) objective on a Deltavision Core microscope (Image Solutions—Applied Precision, GE) attached to a CoolSNAP HQ2 CCD camera. Deconvolution was performed using SoftWoRx Suite 2.0 (Applied Precision, GE). Video microscopy was conducted with the DeltaVision Core microscope as above. Normal growth conditions were maintained throughout the experiment (37°C; 5% CO_2_). Images were recorded at one frame per 7 minutes using SoftWoRx software. Further image processing was performed using ImageJ64 software.

Superresolution microscopy (SR-SIM) was carried out using an ELYRA S.1 microscope (Zeiss) equipped with a Plan Apochromat 63×, 1.4 NA oil immersion lens. Photographs were recorded with CoolSNAP HQ camera (Photometrics) and processed using ZEN Black software (Zeiss). Colocalisation analysis was performed using ZEN Black software. Briefly, a cut mask of only pixels colocalising between the 594 and 488 nm channels above the default threshold was obtained, the resulting Z-stack was then maximally projected into a single image in order to display colocalisation throughout the entire cell.

For statistical analysis of protein distribution, parasites were manually delineated and foci detected using automatic thresholding in ImageJ. G-functions were determined using the Spatial Analysis 2D/3D ImageJ plugin as previously described [36]. Spatial distribution of protein localisation from at least 15 parasites from two independent experiments was determined.

### Transmission electron microscopy

A monolayer of HFF cells were infected with ddMyc-MyoA_tail_ (with or without 1 μM Shld) loxP*gap40* or loxP*gap50*, induced with 50 nM rapamycin for 4 h and fixed with 2.5% glutaraldehyde in 0.1 M phosphate buffer pH 7.4 after the indicated incubation. Samples were processed for routine electron microscopy as described previously [43] and examined in a JEOL 1200EX electron microscope.

### Co-immunoprecipitation and western blot

1 x 10^7^ ddMyc-MyoA_tail_ or ddMyc-GFP parasites were resuspended in 400 μl of Co-IP buffer (50 mM Tris-HCL pH 7.4, 150 mM NaCl, 5 mM EDTA, 1% Triton X-100 with protease inhibitors (cOmplete EDTA-free Mini, Roche)) and incubated on ice for 10 mins. To ensure complete lysis, parasites were subjected to freeze/thaw three times followed by 2 mins of sonication. The lysate was then centrifuged at 14,000 rpm for 10 mins at 4°C and the supernatant removed and incubated with 2.5 μl of anti-Myc (Sigma) antibody for 1 h at 4°C with rotation. 50 μl of Dynabeads (Life Technologies) pre-equilibrated in Co-IP buffer were then added and incubated for a further hour at 4°C with rotation. Dynabeads were then washed four times in 500 μl of co-IP buffer before resuspension in 75 μl of Co-IP buffer and addition of SDS-loading buffer. Samples were incubated at 60°C for 10 mins before being loaded on a precast 12% SDS-PAGE gel. Western blotting was performed as previously described [22].

### Osmotic shock assay

loxP*gap40* parasites were inoculated onto HFF cells and either untreated or induced with 1 μM rapamycin for 24 h before being mechanically released from host cells. Parasites were then spun onto poly-L-lysine coated coverslips at 500 x *g* for 5 min and media replaced with Hanks balanced salt solution (HBSS). Cells were then treated with decreasing concentrations of HBSS diluted in sterile, deionised H_2_O, 1 M sorbitol (in HBSS) or left untreated and incubated at room temperature for 5 mins. The supernatant was then removed and replaced with HBSS with propidium iodide (PI) and incubated at room temperature for 10 mins. This was removed, coverslips washed once with HBSS and fixed and stained as described above. At least 50 cells staining over three fields of view were counted and the proportion positive for PI was determined. Values were normalised to untreated GFP-lacZ parasites [11] and the values are the mean of at least three independent experiments.

### Vacuole size quantification

At indicated time points, loxP*gap40* or *gap40* KOi parasites were fixed, loxP*gap40* parasites were then stained using anti-actin. Images of at least 50 vacuoles per condition were then acquired using a 63 × oil objective on a Zeiss Axioskope 2 MOT+ microscope attached to an Axiocam MRm CCD camera using Volocity software. Images were pre-screened manually and any with vacuoles that appeared to be touching were discarded. Vacuole area was demined using a CellProfiler pipeline [44]. Briefly, images from the green channel were isolated, converted to greyscale and vacuoles were identified as primary objects between 10 and 200 pixels. Objects touching the border were discarded and vacuole area was measured. Results representative of three independent experiments.

### Disruption of GAPM genes by transient CRISPR-Cas9 expression

sgRNA plasmids were synthesised by GenScript (USA), recognition sequences are listed in Table 2. RH Δhxgpt parasites were transiently transfected using an AMAXA 4D Nucleofector (Lonza) with Cas9-YFP and each sgRNA plasmids. Briefly, a total of 10 μg of precipitated plasmid DNA and approximately 9 x 10^5^ parasites were resuspended in 20 μl of Buffer P3 (P3 Primary Cell 4D Nucleofector X kit S (32 RCT), Lonza). Parasites were transfected in a multi-well format using a programme FI-158, transferred to an HFF monolayer and fixed at 24 and 48 h post transfection. Parasites were stained using mouse anti-IMC1 and anti-GAP40 and quantified. Parasites were considered transfected if expressing GFP-Cas9 in the nucleus.

## Supporting Information

S1 Movie *gap40* KOi parasites collapse after initiation of the cell cycle. Parasites were transiently transfected with IMC1-tomato, allowed to express overnight before the addition of 50 nM rapamycin. 4 hours after addition, imaging was initiated and images taken every 7 minutes. The second loop shows only the red channel allowing clearer visualisation of the IMC. Time stamp in mins in upper right corner, scale bar 5 μm.
(AVI)

S2 Movie Normal *T*. *gondii* replication. LoxP*gap40* parasites transiently transfected with IMC1-tomato display normal replication with initiation of daughter cell buds followed by lengthening of the IMC and finally budding of the daughter cells. Scale bar 5 μm.
(AVI)

S3 Movie *gap40* KOi parasites show dissociation between the plasma membrane and the IMC conditions *gap40* KOi parasites were transiently transfected with IMC1-tomato, incubated overnight to allow protein expression then excision was induced. 4 h after the addition of rapamycin, imaging was initiated and images captured every 7 mins. YFP signal was observed to appear outwith the IMC boundaries. The second loop shows only the red channel allowing clearer visualization of the IMC. Time stamp in mins in upper right corner, scale bar 5 μm.
(AVI)

S4 Movie *gap40* KOi parasites show dissociation between the plasma membrane and the IMC. As above but including transmission images to allow the visualisation of the parasite plasma membrane and parasitophorous vacuole. Time stamp in mins in upper right corner, scale bar 5 μm.
(AVI)

S1 Fig Overexpression of MyoA_tail_ does not significantly affect replication of organelles Expression of MyoA_tail_ did not result in major morphological changes of the apicoplast (localised using FNR-RFP) or the Golgi (localised using GRASP-RFP). Although the structure of the mitochondria was affected (localised using HSP60-RFP) this is probably a secondary effect, due to collapse of the IMC. IFA using specific antibodies against rhoptries (ROP5), micronemes (MIC3, M2APro) and dense granules (GRA9) demonstrated that specialised secretory organelles were present although localisation of these structures was affected. Scale bar 10 μm.
(PDF)

S2 Fig ddMyc-MyoA_tail_ is able to immunoprecipitate GAP40. Parasites treated with 1μM Shld-1 for 24 h were lysed and anti-Myc used for immunoprecipitation. ddMyc-MyoA_tail_, but not ddMyc-GFP, was able to immunoprecipitate GAP40. Result is representative of at least four independent experiments.
(PDF)

S3 Fig Excision of the *gap40* gene leads to GAP40 protein levels becoming undetectable. Excision was induced by 50 mM rapamycin and parasites incubated for the indicated time before fixation and staining using anti-GAP40. After induction the majority of parasites express YFP, indicating excision of the *gap40* gene. Although GAP40 is detectable in some vacuoles as long as 40 h post excision, parasites show a severe defect in IMC biogenesis. For a clearer demonstration of the phenotypes we chose images where non-excised controls (loxP*gap40*) are next to *gap40* KOi parasites. Scale bar 10 μm.
(PDF)

S4 Fig A proportion of MLC1 remains associated with the disrupted IMC in the *gap40* KOi. In the loxPgap40 strain, MLC1 and IMC1 are tightly co-localised. However, in the *gap40* KOi parasites, a proportion of MLC1 remains associated with the fragmented IMC (arrows) while the remainder is seen in small vesicles throughout the cytoplasm. Scale bar 5 μm.
(PDF)

S5 Fig Deletion of *gap40* does not initially affect nuclear or major organelle replication. **a**. At 24 h post induction of excision, there is no significant difference in the number of nuclei/vacuole between the parental strain (MG311), *loxPgap40* and *gap40* KOi. Results mean ± standard deviation of three independent experiments. **b**. Golgi replication and segregation was not affected. Parasites were transiently transfected with GRASP-RFP to visualise the Golgi. **c**. Excision of *gap40* did not appear to affect apicoplast (visualised using anti-HSP60) morphology. **d**. The ratio of apicoplasts to nuclei was not significantly affected at 24 h post induction. Results mean of three independent experiments ± standard deviation. **e**. At 24 h post induction of excision, both micronemes (visualised by an antibody against AMA1) and rhoptieries (ROP2/4) are present in affected parasites although both organelles lose their localisation probably due to the loss of parasite morphology. Parasites were transiently transfected with HSP60-RFP, a marker for the mitochondria, before induction and fixation at 24 or 40 h. At both time points, the mitochondrion appeared to expand, however segregation of the organelle appeared abnormal or absent. Scale bar 10 μm.
(PDF)

S6 Fig GAP45 and MLC1 are smoothly distributed over the IMC. After visualising the subpellicular microtubules with anti-acetylated tubulin, neither GAP45 (**a**) nor MLC1 (**b**) were observed to follow the pattern of microtubules. Scale bar 5 μm, detail images scale bar 1 μm. **c**. Statistical analysis demonstrated that the distribution of both GAP45 and MLC1 (blue lines) showed no deviation from that predicted by chance alone (red line, green line indicated 95% confidence limits). Graph representative of at least 15 individual cells from two separate experiments.
(PDF)
