# Supplementary figures and images for: Gliding Associated Proteins Play Essential Roles during the Formation of the Inner Membrane Complex of Toxoplasma gondii
Source: PLoS Pathog. 2016 Feb 4;12(2):e1005403. doi: 10.1371/journal.ppat.1005403 (PMC4742064; doi:10.1371/journal.ppat.1005403)

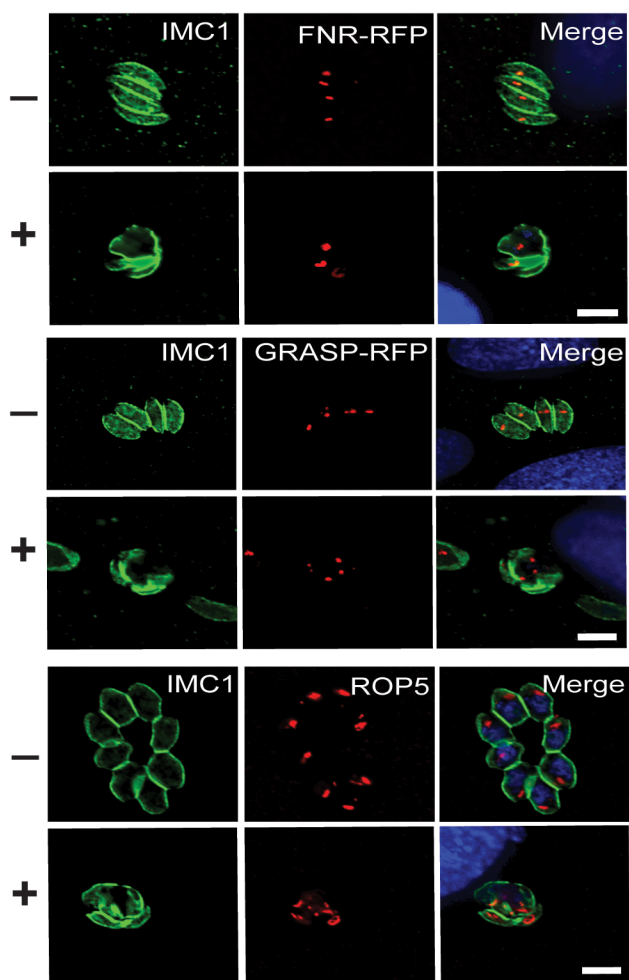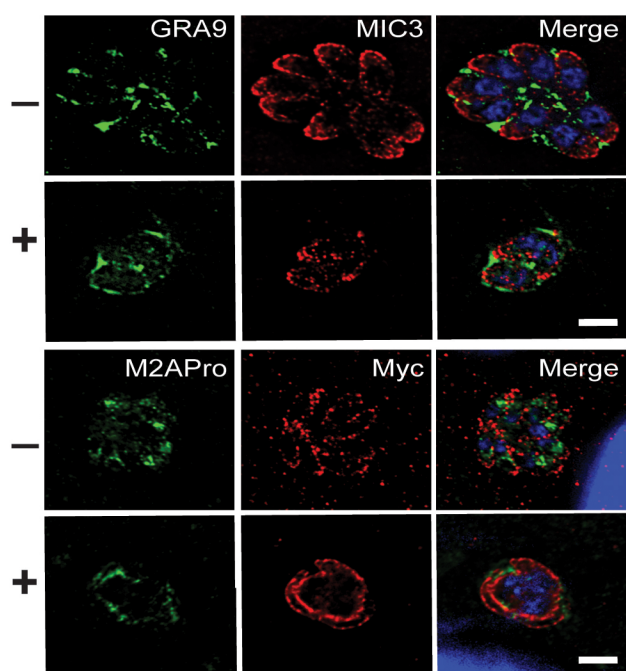

Supplement: S1 Fig — Although the structure of the mitochondria was affected (localised using HSP60-RFP) this is probably a secondary effect, due to collapse of the IMC. IFA using specific antibodies against rhoptries (ROP5), micronemes (MIC3, M2APro) and dense granules (GRA9) demonstrated that specialised secretory organelles were present although localisation of these structures was affected. Scale bar 10 μm. (PDF) [file ppat.1005403.s005.pdf]

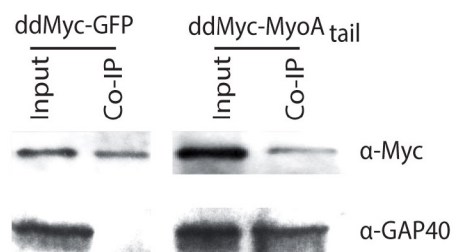

Supplement: S2 Fig — Parasites treated with 1μM Shld-1 for 24 h were lysed and anti-Myc used for immunoprecipitation. ddMyc-MyoAtail, but not ddMyc-GFP, was able to immunoprecipitate GAP40. Result is representative of at least four independent experiments. (PDF) [file ppat.1005403.s006.pdf]

18 h pl

GAP40

YFP

Merge

24 h pl

40 h pl

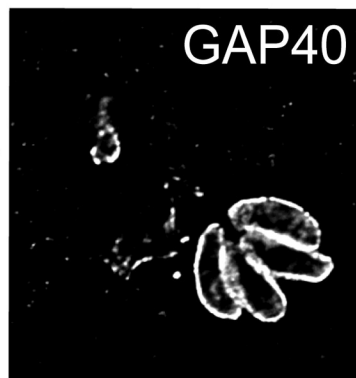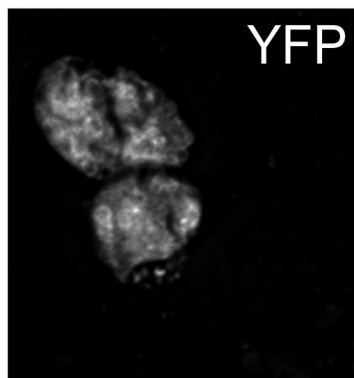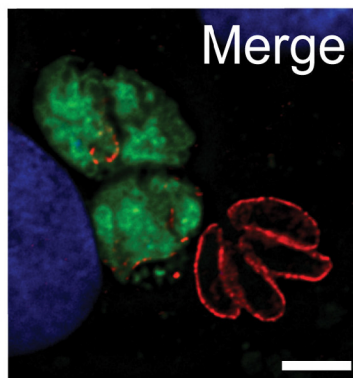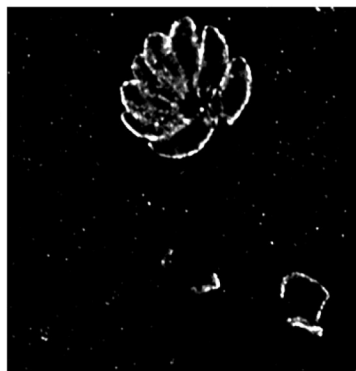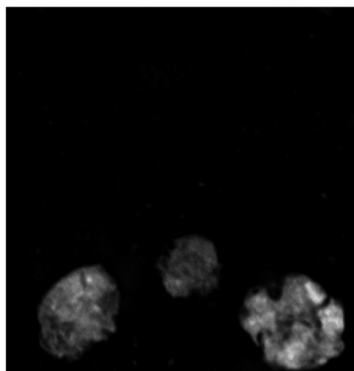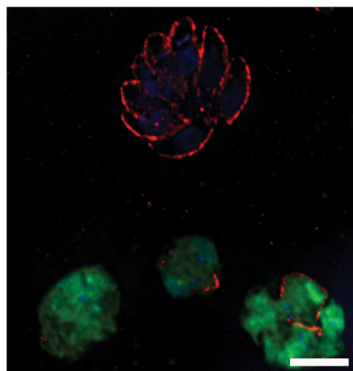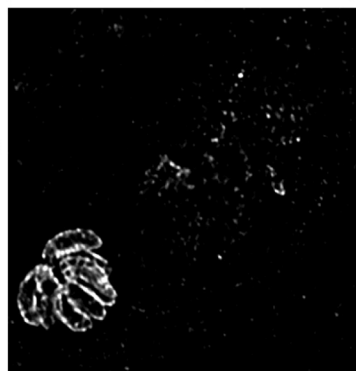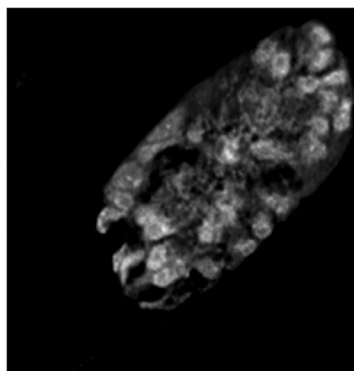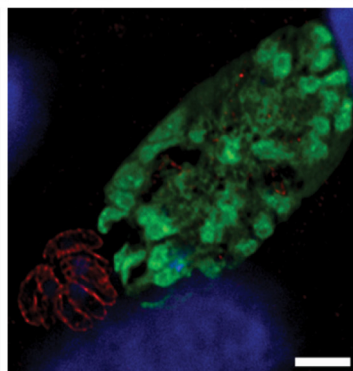

Supplement: S3 Fig — Excision was induced by 50 mM rapamycin and parasites incubated for the indicated time before fixation and staining using anti-GAP40. After induction the majority of parasites express YFP, indicating excision of the gap40 gene. Although GAP40 is detectable in some vacuoles as long as 40 h post excision, parasites show a severe defect in IMC biogenesis. For a clearer demonstration of the phenotypes we chose images where non-excised controls (loxPgap40) are next to gap40 KOi parasites. Scale bar 10 μm. (PDF) [file ppat.1005403.s007.pdf]

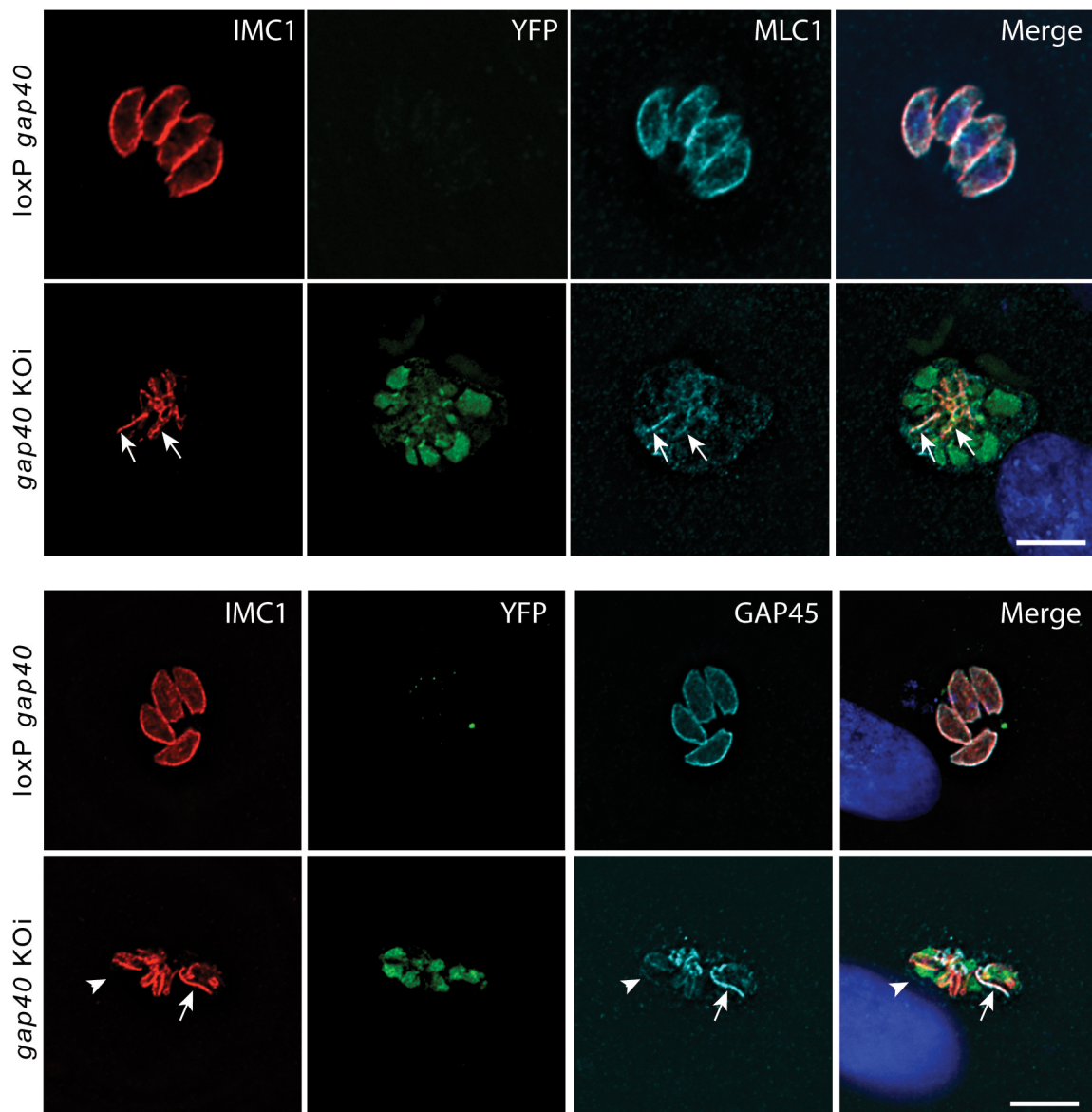

Supplement: S4 Fig — In the loxPgap40 strain, MLC1 and IMC1 are tightly co-localised. However, in the gap40 KOi parasites, a proportion of MLC1 remains associated with the fragmented IMC (arrows) while the remainder is seen in small vesicles throughout the cytoplasm. Scale bar 5 μm. (PDF) [file ppat.1005403.s008.pdf]

**a**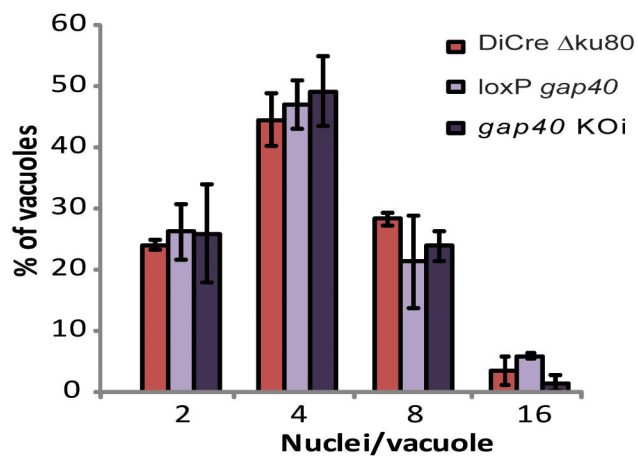**b**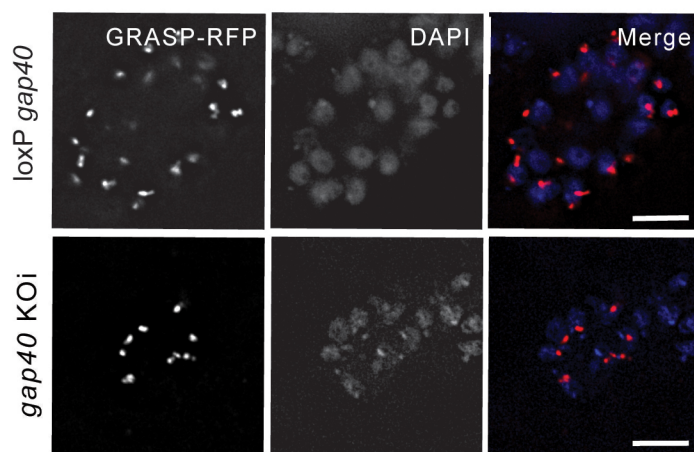**c**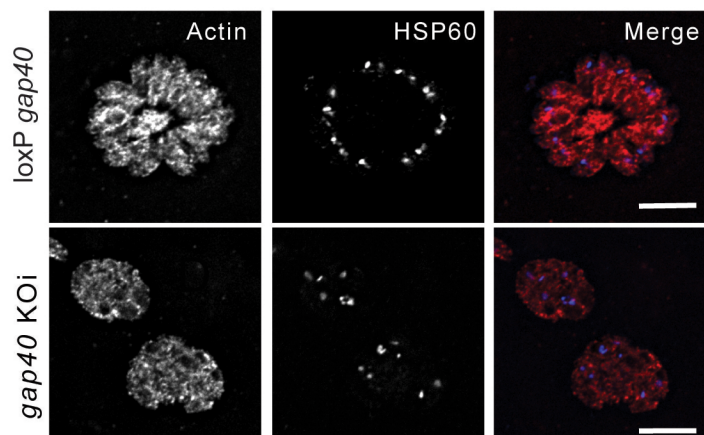**d**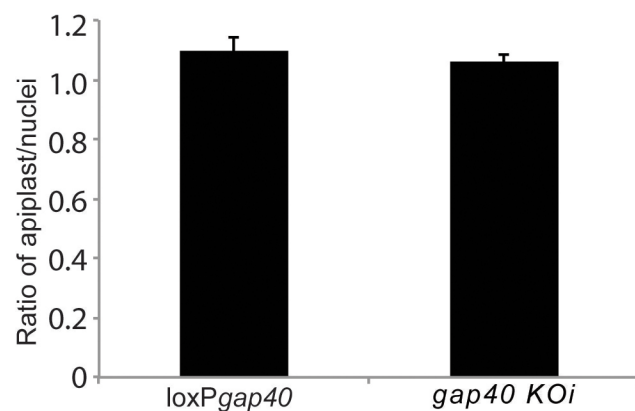**e**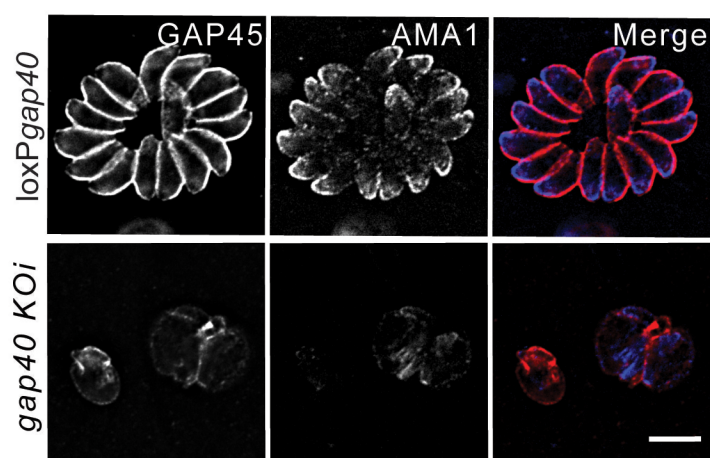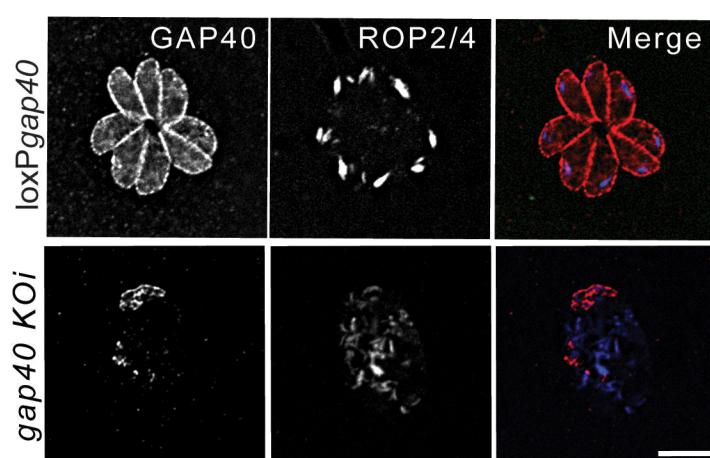**f**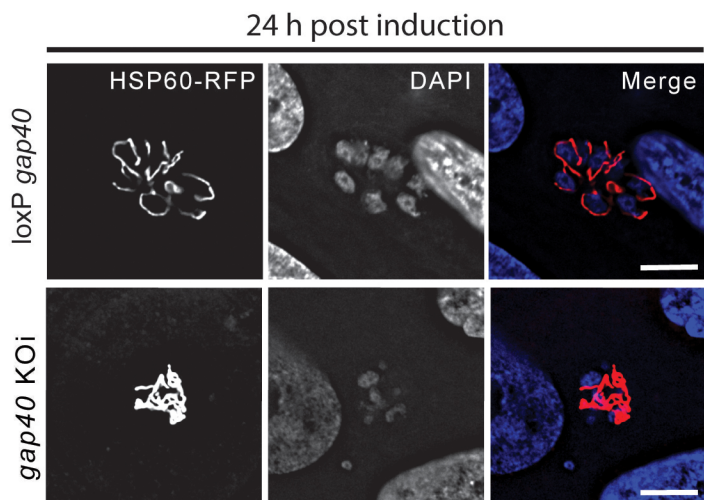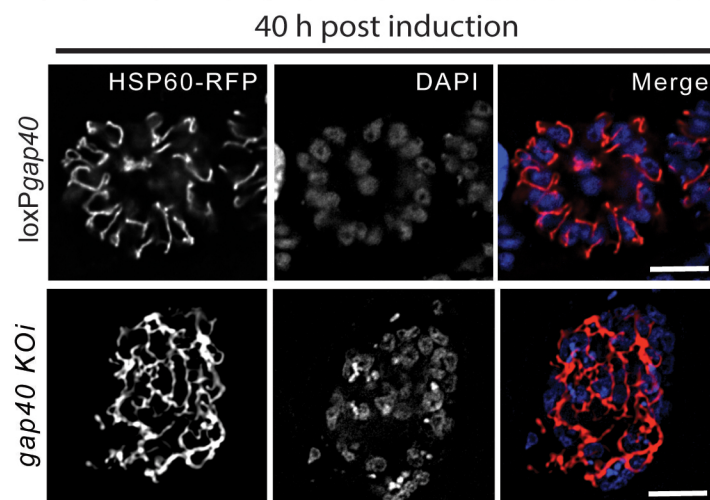

Supplement: S5 Fig — a. At 24 h post induction of excision, there is no significant difference in the number of nuclei/vacuole between the parental strain (MG311), loxPgap40 and gap40 KOi. Results mean ± standard deviation of three independent experiments. b. Golgi replication and segregation was not affected. Parasites were transiently transfected with GRASP-RFP to visualise the Golgi. c. Excision of gap40 did not appear to affect apicoplast (visualised using anti-HSP60) morphology. d. The ratio of apicoplasts to nuclei was not significantly affected at 24 h post induction. Results mean of three independent experiments ± standard deviation. e. At 24 h post induction of excision, both micronemes (visualised by an antibody against AMA1) and rhoptieries (ROP2/4) are present in affected parasites although both organelles lose their localisation probably due to the loss of parasite morphology. Parasites were transiently transfected with HSP60-RFP, a marker for the mitochondria, before induction and fixation at 24 or 40 h. At both time points, the mitochondrion appeared to expand, however segregation of the organelle appeared abnormal or absent. Scale bar 10 μm. (PDF) [file ppat.1005403.s009.pdf]

**a**

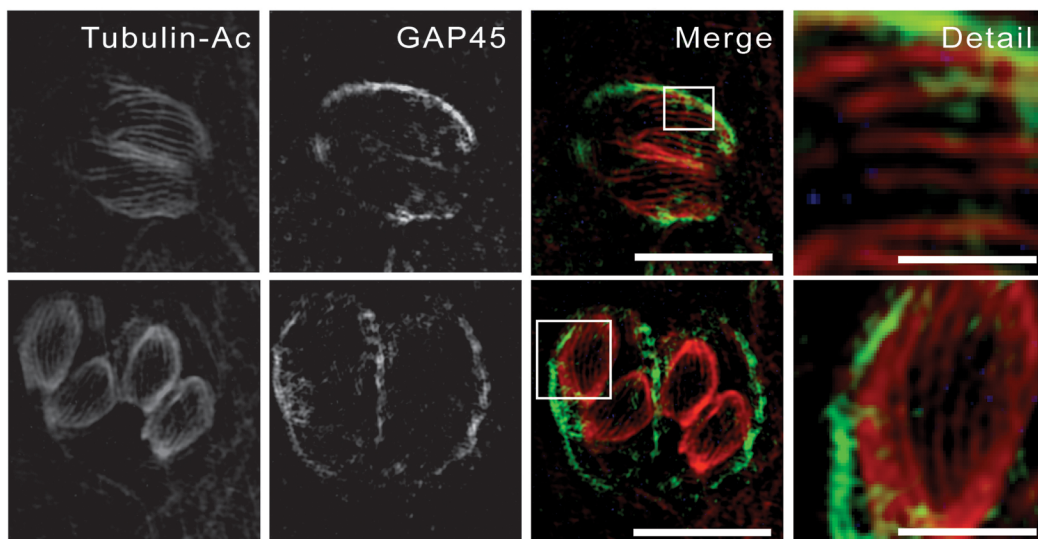

**b**

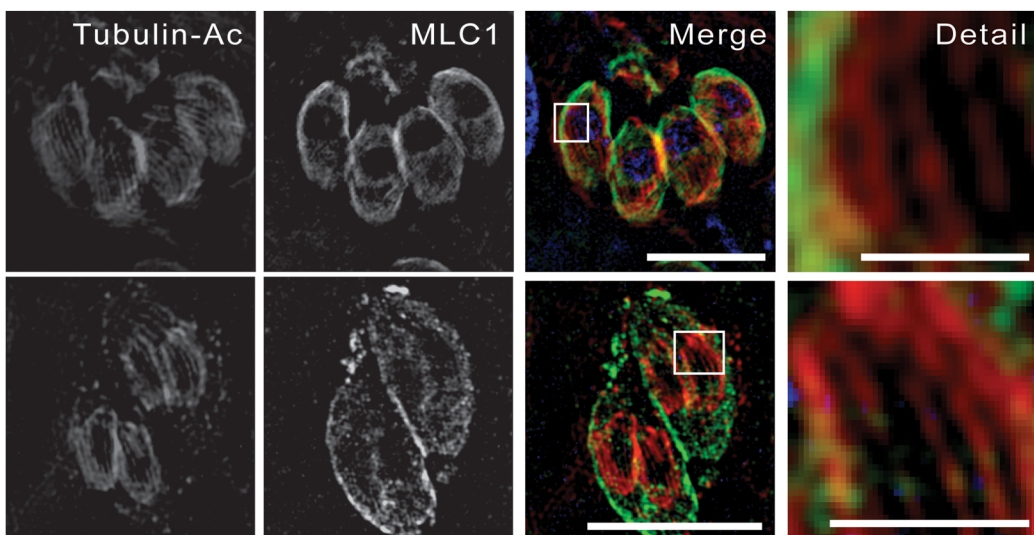

**c**

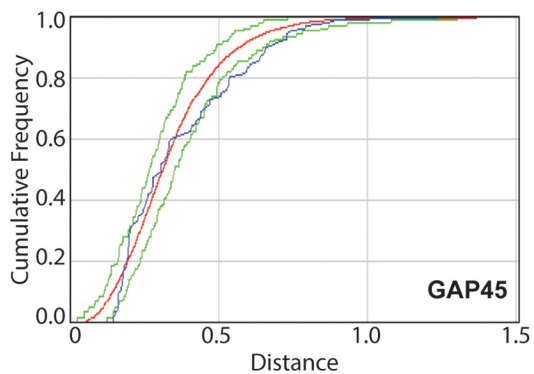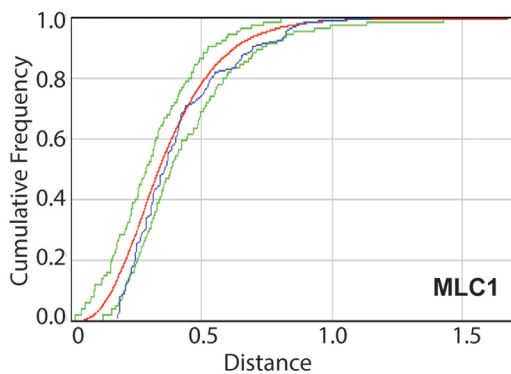

Supplement: S6 Fig — After visualising the subpellicular microtubules with anti-acetylated tubulin, neither GAP45 (a) nor MLC1 (b) were observed to follow the pattern of microtubules. Scale bar 5 μm, detail images scale bar 1 μm. c. Statistical analysis demonstrated that the distribution of both GAP45 and MLC1 (blue lines) showed no deviation from that predicted by chance alone (red line, green line indicated 95% confidence limits). Graph representative of at least 15 individual cells from two separate experiments. (PDF) [file ppat.1005403.s010.pdf]
